# The Effect of Voluntary Staying at Home on Japanese Female Suicide During the COVID‐19 Pandemic

**DOI:** 10.1002/hec.70078

**Published:** 2026-01-11

**Authors:** Yoko Ibuka, Haruo Kakehi, Ryuki Kobayashi, Ryo Nakajima

**Affiliations:** ^1^ Faculty of Economics Keio University Tokyo Japan; ^2^ Graduate School of Economics Keio University Tokyo Japan; ^3^ Department of Economics University of Wisconsin–Madison Madison Wisconsin USA

**Keywords:** females, mental health, shift‐share IV, specification curve analysis, voluntary lockdown

## Abstract

In Japan, female suicide increased during the COVID‐19 pandemic. This study evaluated how pandemic‐related home confinement affected female suicide. We employed a shift‐share instrumental variable design to assess whether differential exposure to the pandemic caused changes in suicide incidence. We found that suicide increased among females under 20 years of age as more people stayed at home. Counterfactual analyses showed that at least 35% of these suicides were attributed to home confinement. Our results suggest that a substantial part of the suicide increase among young females was driven by lifestyle changes during the pandemic.

## 1 Introduction

Suicide is a serious public health problem with detrimental effects on both individuals and society. Japan has long had one of the highest suicide rates among the Organization of Economic Co‐operation and Development countries. The economic cost of suicide amounted to 21.7 billion USD in 2010 (IPSS 2010).^1^ The COVID‐19 pandemic, which began in 2020, further increased the risk of suicide (Pirkis et al. 2022). Worryingly, after a decade of decline attributable to the comprehensive suicide‐prevention initiatives introduced in 2006, Japan's suicide rate again started rising during the COVID‐19 pandemic. Extant studies show a clear and consistent pattern of increased suicide during the pandemic, especially among young people and women (Tanaka and Okamoto 2021; Sakamoto et al. 2021; Ueda et al. 2021). With fertility already at historic lows, the rise in suicides among young women is a deepening social crisis in Japan.

**FIGURE 1:**
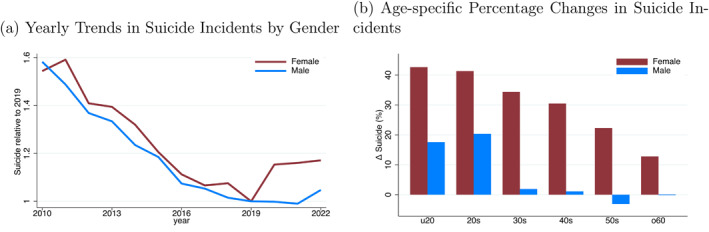
Suicide trends in Japan: impact of COVID‐19. (a) Yearly trends in suicide incidents by gender. (b) Age‐specific percentage changes in suicide incidents. Panel (a) displays the long‐term yearly trend in the number of suicide incidents among men and women in Japan from 2010 to 2022, with 2019 as the base year. Panel (b) shows the percentage change in the number of suicide incidents among men and women after COVID‐19 compared with that before COVID‐19, broken down by age group. The sample period spans from April 2019 to March 2021. We compare cases from April 2020 to March 2021 with those from April 2019 to March 2020.

Figure 1a shows the annual changes in suicide rates by gender since 2010. While a decline was observed until 2019, this trend reversed for females in 2020. Furthermore, Figure 1b shows that the post‐pandemic increase in suicide is the greatest among young females and decreases with age. This contrasts with the pattern of suicide increases caused by a traditional macroeconomic shock, where males, particularly those of working age, are more strongly affected by economic circumstances (Koo and Cox 2008; Chen et al. 2015; Huikari et al. 2019). The difference between the affected population in the pandemic and that in past macroeconomic shocks likely suggests the influence of another factor distinct from traditional economic problems.

One such factor may be the public‐health dimension of the pandemic. It can raise the risk of mental health problems not only among those who are infected, but also among those who are not through direct and indirect channels (Penninx et al. 2022). Studies have identified infectious‐disease‐related factors affecting psychological well‐being during the 2003 severe acute respiratory syndrome (SARS) outbreak, 2009 H1N1 pandemic, and 2022–2023 monkeypox outbreaks (Cowling et al. 2010; Le Forestier et al. 2024; Yip et al. 2010). Among these factors, social disconnection has emerged as a major risk factor for the general population, including those who were not infected. For instance, SARS‐related suicide deaths were linked to social isolation, one of the two primary factors, alongside the fear of infection (Yip et al. 2010). In the COVID‐19 pandemic context, early public health responses included strong social distancing measures such as lockdowns and stay‐at‐home orders. Although the intensity of such policies varied by country, most developed countries, including Japan, kept large segments of society at home in the early stages of the pandemic.^2^ The World Health Organization (WHO) warned that social disconnection during the pandemic could increase the risk of mental illness. One study validated this finding, showing a positive association between the stringency of social distancing policies and prevalence of mental health problems (Aknin et al. 2022).

Here, we explore how the extent of staying at home affects suicide to understand the mechanism underlying the most pronounced increase in suicide among young females in Japan. Studies examined the impact of social distancing on various outcomes, including mental health, leveraging regional differences in the degree and/or timing of stay‐at‐home policies (Baek et al. 2021; Altindag et al. 2022; Serrano‐Alarcón et al. 2022). We focus on the influence of the voluntary nature of staying at home on suicide and assess the effect of home confinement among compliers. This is important because voluntary social distancing, as opposed to policy‐induced social distancing, represents a substantial portion of the observed behavioral responses (Yan et al. 2021). This matters even more in the Japanese context, where curfews imposed under the state of emergency had no legal binding force through fines or arrests; consequently, the extent to which people actually stayed at home was endogenous.

To address the endogeneity problem of staying‐at‐home behavior, we employ a shift‐share instrumental variable (IV) design. Specifically, using municipality‐level data on suicide, our analysis exploits the geographical variation in exposure to the pandemic and tests to examine whether a *differential* exposure to the shock leads to *differential* changes in the suicide incidence at the municipality level. We employ an IV strategy in which a change in stay‐at‐home behavior in a municipality is predicted by pre‐pandemic commuting behavior.

This study contributes to the literature in three ways. First, we add to the economics literature on mental health by examining physical disconnection from society as a risk factor in suicide. To date, the economic literature on psychological well‐being and suicide has primarily focused on economic factors (Hamermesh and Soss 1974; Darity and Goldsmith 1996; Ruhm 2000), indicating that working‐age males are most vulnerable to macroeconomic shocks such as financial crises (Chen et al. 2009; Chen et al. 2015; Marcotte and Hansen 2024). Second, we reveal individual patterns of home confinement as the mechanism driving the increase in suicide rates during the COVID‐19 pandemic. Thus far, several economic and public health studies have shown an elevated risk of the symptoms of depression and other mental disorders, an increase in calls to mental health helplines, suicide attempts, and self‐harm during the pandemic (Brodeur et al. 2021; Brülhart et al. 2021; Dubé et al. 2021; Vahratian et al. 2021; John et al. 2020). However, few studies have analyzed the underlying mechanisms of the adverse mental health effects. Understanding the pathways via which suicide rates increase has important implications for public policies on both infectious disease control and suicide prevention. Additionally, quantifying the social costs of public health responses during the pandemic in terms of human lives is important. Third, we employ a shift‐share IV design to obtain the causal impact (Borusyak et al. 2025). Mobility tends to be endogenous in terms of the outcome variables related to individual decisions. Addressing this issue is an important empirical consideration.

## 2 Empirical Model

### 2.1 Setup

To estimate the effect of staying at home on suicide incidence, we use the following structural equation:

(1) $$\ln\left(1+Suicide_{mt}\right)=\alpha_{m}+\delta \times t+\beta StayHome_{mt}+\varepsilon_{mt},$$

where m indexes the municipalities and t indexes the time period. The outcome variable of interest is the natural logarithm of one plus the number of suicide cases, $Suicide_{mt}$, in the municipality m in period t.^3^ We consider two time periods: a pre‐COVID‐19 period for t=0 (from April 2019 to March 2020) and post‐COVID‐19 period for t=1 (from April 2020 to March 2021).^4^

The explanatory variable of our focus is the extent of staying at home, $StayHome_{mt}$, of residents in municipality m in period t. It is potentially endogenous and influenced by unobserved confounding factors such as area‐specific psychological traits and environmental conditions. For instance, certain regions may exhibit an elevated incidence of mental health challenges due to factors, such as climatic conditions or geographical features. These factors may simultaneously affect both suicidal and stay‐at‐home behaviors. We control for municipality‐specific fixed effects, $\alpha_{m}$ and common time trends, δ×t.

In practical applications, when addressing the municipality‐specific fixed effects within a panel data structure, a common approach is to employ a first‐difference specification designed to eliminate the influence of the municipality fixed effect, $\alpha_{m}$. This approach is expressed as follows:

(2) $$\Delta \;\ln\left(1+Suicide_{m}\right)=\delta +\beta \Delta StayHome_{m}+\Delta \varepsilon_{m},$$

where Δ represents the first‐differencing operator.

To mitigate endogeneity concerns regarding $StayHome_{mt}$ in Equation (1) or $\Delta StayHome_{m}$ in Equation (2), we employ a shift‐share instrument. This method introduces an additional dimension, distinct from both the cross‐sectional and time dimensions, aimed at reducing the influence of potential confounders on our causal estimates. Specifically, we express $StayHome_{mt}$ as the average proportion of time residents spent at home in municipality m during period t as follows:

$$StayHome_{mt}=\sum_{i\in N_{m}}\left(\frac{1}{n_{m}}\right)s_{it}.$$

Here, i represents residents, $N_{m}$ denotes the set of municipality m residents, $n_{m}$ indicates the total resident count, and $s_{it}$ signifies the proportion of time a resident i spends at home during the period t. Importantly, $s_{it}$ is influenced by municipality‐specific psychological and environmental factors, making $StayHome_{mt}$ an endogenous variable within the structural equation. In the context of the shift‐share IV approach, $1/n_{m}$ represents a share‐related factor, whereas $s_{it}$ corresponds to a shift‐related element. Consequently, the endogenous variable is articulated as the inner product of these share and shift components.

Utilizing the endogenous variable's inner product structure, we construct a shift‐share instrument that categorizes residents into K types based on their stay‐at‐home behavior. Assuming similar stay‐at‐home propensities among residents of each type k, we define $s_{\mathrm{ikt}}$ as $s_{\mathrm{ikt}}=g_{kt}+\xi_{\mathrm{ikt}}$. Here, $g_{kt}$ indicates the average proportion of time that type k residents spend at home in period t, whereas $\xi_{\mathrm{ikt}}$ captures the idiosyncratic deviation from the typical stay‐at‐home propensity for each resident i of type k in period t. Note that the individual‐specific term $\xi_{\mathrm{ikt}}$ may be correlated with various confounding factors in municipality m.

Accordingly, we reformulate the stay‐at‐home variable specific to each municipality as follows:

(3) $$\begin{array}{c} StayHome_{mt}=\sum_{k\in K}\left(\frac{n_{mk}}{n_{m}}\right)g_{kt}+\sum_{k\in K}\sum_{i\in N_{mk}}\left(\frac{1}{n_{m}}\right)\xi_{\mathrm{ikt}}, \end{array}$$

where $N_{mk}$ is the cohort of type‐k residents in municipality m and $n_{mk}$ is their number. The first term, representing the shift‐share instrument $ShiftShare_{mt}$, isolates the exogenous variation in $StayHome_{mt}$ from the endogenous individual component related to $\xi_{\mathrm{ikt}}$. With $z_{mk}=n_{mk}/n_{m}$ denoting the proportion of type‐k residents in m, the shift‐share instrument, $ShiftShare_{mt}=\sum_{k}z_{mk}g_{kt}$, comprises two elements: the share component $\left(z_{mk}\right)$, which is predetermined and governing exposure to shocks, and shift component $\left(g_{kt}\right)$, which is a common trend among type‐k residents across municipalities.

### 2.2 Shift‐Share IV: Two Types

To illustrate the shift‐share IV estimation, we use a simplified scenario to focus on the differential impact of COVID‐19 on two types of residents: *commuters* and *noncommuters*. Suppose that noncommuters (represented as k=0) predominantly stayed at home, whereas commuters (represented as k=1) were mostly away before the pandemic started. Emergency declarations in the post‐COVID‐19 period led both groups to largely stay at home. This change is captured by setting the following average stay‐at‐home time proportions: $g_{10}=0$ and $g_{00}=g_{01}=g_{11}=1$. Thus, for any resident i of type k in municipality m, $ShiftShare_{mt}=\sum_{k}z_{mk}g_{kt}=\left(t-1\right)z_{m1}+1$, where $z_{m1}$ is the proportion of commuters in municipality m in the pre‐COVID‐19 period. In a time‐difference form, given that $\Delta g_{1}=1$ and $\Delta g_{0}=0$, we obtain a simple expression as $\Delta ShiftShare_{m}=z_{m1}$.

This approach, often referred to as the *differential exposure design*, exploits the stark contrast in the stay‐at‐home duration triggered by COVID‐19.^5^ Noncommuters maintain their longer stay‐at‐home tendencies, whereas commuters newly experience working from home under the new state of emergency order. Therefore, the “treatment” municipalities with more commuters experience greater COVID‐19 exposure than “control” municipalities with fewer commuters. The effect is gauged by the commuter population's relative change, aligning with the difference‐in‐differences (DiD) methodology in a regression framework, albeit with a continuous treatment variable.

**FIGURE 2:**
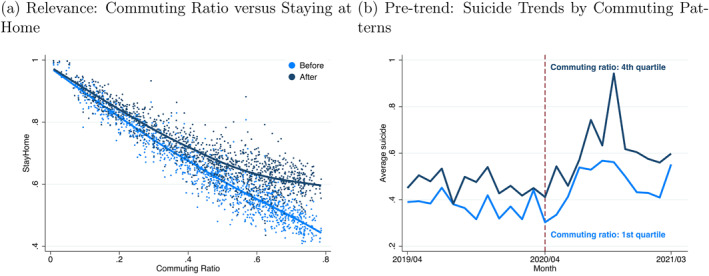
Relevance and pre‐trend: two‐type case. (a) Relevance: commuting ratio versus staying at home. (b) Pre‐trend: suicide trends by commuting patterns. Panel (a) depicts the relationship between the commuting ratio $z_{m1}$ and stay‐at‐home variable $StayHome_{mt}$ at two distinct time points, t=0 and t=1. Panel (b) compares the average number of suicide incidents between the first and fourth quartiles of the commuting ratio $z_{m1}$, covering the sample period from April 2019 to March 2021. A vertical dashed line marks April 2020, dividing the analysis into two periods: Pre‐COVID‐19 (prior to April 2020) and Post‐COVID‐19 (from April 2020 onwards). The commuting ratio's definition can be found in Supporting Information S1: Appendix C.

Two key assumptions must be satisfied to estimate an unbiased treatment effect. First, the shift‐share IV must accurately represent the stay‐at‐home variable, a necessity known as the *relevance* condition, to prevent bias from weak IVs. This entails a significant correlation between $\Delta StayHome_{m}$ and $\Delta ShiftShare_{m}$, or equivalently, between $\Delta StayHome_{m}$ and the commuting ratio $z_{1m}$. Figure 2a displays the scatter plots that link stay‐at‐home rates to commuting ratios, illustrating that municipalities with higher commuting ratios typically have lower stay‐at‐home rates both before and after the COVID‐19 outbreak. Notably, the variation in stay‐at‐home rates across the periods (depicted by the vertical distance between the dark and light blue dots in the figure) is more pronounced in municipalities with higher commuting ratios. This pattern suggests a positive correlation between the change in stay‐at‐home rates over the two periods and the commuting ratio, reinforcing the validity of the relevance assumption with empirical evidence despite the depiction's graphical nature.

Second, consistent with the DiD methodology, the treatment and control groups must exhibit *parallel trends* under a counterfactual scenario. Figure 2b, which compares the average number of female suicide incidents across municipalities with different commuting ratios, shows nearly parallel pre‐pandemic trends but a noticeable increase in suicides in areas with higher commuting ratios in the post‐pandemic period, resulting in a trend divergence. The figure visually demonstrates the similarity of the pre‐trends. Formal statistical pre‐trend tests are detailed in Section 4.2.

### 2.3 Shift‐Share IV: Multiple Types

In the canonical case, a single municipal characteristic—the commuting ratio—defines the shift‐share instrument. The two‐type example provides a useful, intuitive illustration of the exposure design. However, as highlighted by Christian and Barrett (2017) and Jaeger et al. (2020), exclusively relying on variations among a limited number of types may compromise the estimation due to the presence of potential confounding factors. To mitigate these issues, we employ an expanded‐type shift‐share instrument that exploits the variation in a municipal attribute across different types. This enhancement allows for a more robust identification of variations. Furthermore, this instrument facilitates the utilization of the diagnostic tools proposed by Goldsmith‐Pinkham et al. (2020).

In our expanded analysis, we categorize residents by their commuting time, assigning a commuting time of zero to those who do not commute, referred to as noncommuters. As detailed in Section 3, our empirical analysis identifies six specific commuting time brackets. Each bracket, denoted as k, is assumed to exhibit a uniform propensity for staying at home. Noncommuters with zero commuting time are expected to be more likely to stay at home during the pre‐pandemic period. Conversely, individuals with longer commuting times are likely to spend more time away due to the distance to their workplaces; thus, they are presumed to have a lower propensity to stay at home. This approach enables us to discern variations in stay‐at‐home behavior across different commuter groups, moving beyond the binary distinction between commuters and noncommuters. Specifically, for noncommuters (k=0), we set $g_{0t}=1$ for both the pre‐ and post‐COVID‐19 periods (t=0 and t=1, respectively), in the same way as in the two‐type case. Conversely, for commuters (k=1…,K), the pre‐COVID‐19 stay‐at‐home rates differ across commuting categories. In the post‐COVID‐19 period, influenced by the state of emergency orders, the average work‐from‐home duration increased. This results in $g_{k0}\leq g_{k1}$ across all commuting types k.

The impact of emergency declarations and commuting restrictions affected commuter types to varying degrees. For instance, medium‐ and long‐distance commuters were likely to experience a more significant shift toward staying at home than noncommuters and those with shorter commutes. Consequently, the time change in the stay‐at‐home propensity, represented by $\Delta g_{kt}=g_{k1}-g_{k0}$, shows distinct patterns across commuter types k. This variation underscores the diverse effects of COVID‐19‐related measures on individuals' commuting habits and work‐from‐home dynamics.

Expanding on the analysis with multiple commuter types, our empirical model comprises the following equations.

The structural equation is given by:

(4) $$\Delta \;\ln\left(1+Suicide_{m}\right)=\delta +\beta \Delta \hat{StayHome_{m}}+\Delta \varepsilon_{m}.$$

The estimated variable, $\Delta \hat{StayHome_{m}}$, is derived from the following first‐stage regression:

(5) $$\Delta StayHome_{m}=\delta^{f}+\pi \Delta ShiftShare_{m}+\Delta \varepsilon_{m}^{f}.$$

The first‐time differenced shift‐share instrument is defined as

(6) $$\Delta ShiftShare_{m}=\sum_{k=1}^{K}z_{mk}\times \Delta g_{k},$$

where $z_{mk}$ represents the proportion of commuters of type k in municipality m before COVID‐19 and $\Delta g_{k}$ indicates the first time‐difference stay‐at‐home propensity for type k commuters, a type‐specific common trend applicable to all municipalities. Finally, the reduced‐form regression is as follows:

(7) $$\Delta \;\ln\left(1+Suicide_{m}\right)=\delta^{r}+\sum_{k=1}^{K}\gamma \left(z_{mk}\times \Delta g_{k}\right)+\Delta \varepsilon_{m}^{r}.$$

In this case, γ=β×π.

Our identification strategy relies on the assumption of share exogeneity, yielding the following exclusion restriction:

(8) $$E\left(z_{mk}\Delta \varepsilon_{m}\right)=0\;\text{for}\;k=1,\text{…},K.$$

This condition implies that $z_{mk}$, as a share, serves as a single instrument. The shift‐share IV is a linear combination of shares, with each share weighted by a shift. The shift‐share estimator $\hat{\beta }$ obtained in this system is the weighted sum of just‐identified IV estimators ${\hat{\beta }}_{k}$, each using a separate share as an instrument (Goldsmith‐Pinkham et al. 2020). These weights, known as *Rotemberg weights*, are particularly useful when multiple instruments are employed. They indicate how much misspecification in each instrument feeds into the estimator's overall bias. The larger an instrument's weight, the more its errors distort the estimate. By highlighting the most influential instruments, Rotemberg weights can help researchers refine their instrument set and bolster the credibility of their results.

## 3 Data

Our analysis is based on municipality‐level data. In Japan, municipalities are the smallest units of local government, and consist of cities, towns, and villages. Each municipality has its own mayor and assembly, and is responsible for local services such as education, welfare, and infrastructure. Japan had 1902 municipalities as of March 2020.^6^ Population and land area vary widely.^7^ As explained below, our analysis focuses on 1241 municipalities, including all cities, as well as those towns and villages with populations of 15,000 or more.

### 3.1 Suicide

We use monthly suicide statistics compiled by the National Police Agency and released by the Ministry of Health, Labor and Welfare (MHLW) (MHLW 2021).^8^ The data collected on incidents at the municipal level is based on the location of residence and are aggregated every month. The data include detailed information on biological sex and age groups, in 10‐year age increments up to 79, and aggregated data for those 80 years or older.^9^

As illustrated in Figure 1a, the suicide rate increased between 2019 and 2020. Crucially, the increase was more pronounced among women. In addition, this trend was not uniform across all age groups in either women or men. Figure 1b shows the percentage change in national suicide incidents by age group, with a more pronounced increase particularly observed among young individuals. Notably, the data indicate an approximately 42.7% increase among females under 20 years of age after the COVID‐19 outbreak.

### 3.2 Stay‐at‐Home Variable

We measure the extent of stay‐at‐home behavior in each municipality during the pre‐ and post‐COVID‐19 periods using mobile phone location data. The data are provided by Agoop, a subsidiary of one of Japan's major mobile phone companies. These data offer hourly population counts for each municipality. These counts are derived from users' time spent in each municipality and adjusted for demographic characteristics.^10^ Agoop's data are available from January 2019 onward, and cover individuals aged 15 and above.

The stay‐at‐home variable for a municipality m in period t is defined as the ratio of the municipality's *daytime population* to its *nighttime population* during that period. The daytime population is averaged using data from 11 a.m. to 2 p.m., and the nighttime population is calculated based on data from 1 a.m. to 4 a.m. This variable measures daily shifts in the resident population, highlighting the contrast between daytime and nighttime numbers. As residents typically stay at home at night, the nighttime population tends to be larger than its daytime counterpart. Consequently, the values of the stay‐at‐home variable range from 0 to 1, with higher values indicating lesser outflow from the municipality and a greater inclination to staying at home during the daytime. Detailed explanations and observable fluctuations in the stay‐at‐home variable are in Supporting Information S1: Appendix Figure A2.

The Agoop data provide valuable insights into residents' movements between municipalities.^11^ Although these data are aggregated at the municipal level and the analysis does not include the detailed time spent at home found in studies based on SafeGraph data, the data still serve as a reliable indicator of stay‐at‐home behavior.^12^ In Supporting Information S1: Appendix Section A.3, we establish the reliability of our stay‐at‐home variable, computed from the Agoop data, by demonstrating its correlation with the Google Mobility Index, which is designed to measure the time spent at home.^13^ This correlation reinforces the effectiveness of our stay‐at‐home measure as a valuable proxy for assessing stay‐at‐home behaviors.

**FIGURE 3:**
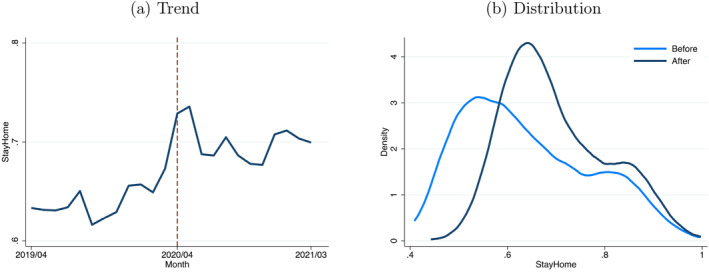
Stay‐at‐home trends and distribution before and after COVID‐19. (a) Trend. (b) Distribution. Panel (a) shows the trend of the stay‐at‐home variable between April 2019 and March 2021, with the lines indicating the average. A vertical dashed line marks April 2020. Panel (b) shows the distribution of the stay‐at‐home variable. Before COVID‐19 refers to the period between April 2019 and March 2020, while after COVID‐19 refers to the period between April 2020 and March 2021. The stay‐at‐home variable is conceptually defined by Equation (3).

The outbreak of COVID‐19 and subsequent declaration of the state of emergency significantly impacted the stay‐at‐home behavior of people across Japan. Figure 3a shows the national average change in the stay‐at‐home variable over time. A notable increase was observed in April 2020, immediately following the outbreak of the pandemic. The index remained above pre‐pandemic levels throughout 2020 and 2021, with some observable seasonal variations. Furthermore, the extent of the shift to stay‐at‐home behavior varied across regions after the COVID‐19 outbreak. Figure 3b shows the distribution of the stay‐at‐home variable before and during the pandemic. After the outbreak of the pandemic, the distribution underwent two significant changes: a shift to the right, indicating a nationwide increase in stay‐at‐home behavior, and a narrowing of the distribution, indicating lower variability among municipalities. Although stay‐at‐home behavior generally increased after the pandemic's outbreak, the degree of this increase was not uniformly distributed across municipalities.

### 3.3 Commuting Time and Work From Home

Our shift‐share IV comprises two components. First, individuals are classified into K categories based on their commuting time. The proportion of residents in different commuting time brackets within a municipality during the pre‐COVID‐19 period forms the share $\left(z_{mk}\right)$ of the IV. Second, the nationwide average tendency of individuals who worked from home in each commuting time bracket before and after COVID‐19 indicates the shift $\left(g_{kt}\right)$.

To determine the share component, we divide the residents into six categories based on their commuting duration. Specifically, k=0 corresponds to noncommuters with zero‐minute commuting time; k=1 represents commuters traveling up to only 30 min; k=2, k=3, and k=4 represent mid‐distance commuters with commuting times of more than 30, and equal to or less than 60 min (30–60 min), more than 60, and equal to or less than 90 min, and more than 90, and equal to or less than 120 min, respectively; and k=5 represents long‐distance commuters who travel more than 120 min.

We base this classification on data from the 2018 Housing and Land Survey of Japan conducted by the Statistics Bureau. The survey collected information on the commuting habits of the household heads. The survey sample comprises individuals who live in all cities and wards, and counties with populations of 15,000 or more.^14^ Our analysis incorporates 1241 out of the 1902 municipalities for which commuting time data are available. Since the 661 out‐of‐sample municipalities account for only 3.4% of Japan's total population, their exclusion has minimal impact on the sample's representativeness and does not materially affect the analysis. Furthermore, Supporting Information S1: Appendix Table B2 compares the in‐sample and out‐of‐sample municipalities to illustrate their differences. The excluded sample has a lower mean commuting ratio. Therefore, it is less exposed to the shock, making it less likely that their exclusion biases the estimated impact. We also conduct robustness checks to assess whether trends driven by municipal attributes can affect our estimates.

Supporting Information S1: Appendix Table B1 shows that 46% of household heads commute to municipalities other than their own, although this rate ranges from less than 1%–78% across municipalities. Furthermore, Supporting Information S1: Appendix Table B3 indicates that the most common commuting time is 0–30 min, with an average of 59% of household heads in this category. This also substantially varies: In the municipality with the lowest share, only 14% commute for less than 30 min. Meanwhile, in the municipality with the highest share, the figure reaches 93%. Because the longest‐distance category—commuters traveling more than 120 minutes—accounts for less than 1% of the sample, we construct an alternative IV that merges the two longest travel‐time categories and use it for the robustness check.

Regarding the shift components $g_{kt}$ for each k commuter type, we use the propensity to *work from home* as a proxy for the inclination to stay at home, reflecting the significant adoption of work‐from‐home practices during the COVID‐19 pandemic, as documented by Okubo (2021),  (2022). In Japan, the state of emergency issued in response to COVID‐19 did not impose legal penalties for noncompliance with stay‐at‐home measures. Instead, employers made voluntary requests to employees to work from home. Consequently, some workers continued commuting during the pandemic and not everyone shifted uniformly to remote work, even amid the rise in infections in late 2020. Thus, changes in stay‐at‐home behavior largely mirrored the adoption of work‐from‐home practices. Importantly, work‐from‐home adoption varied across industries, occupations, regions, and commuting times. Our identification strategy exploits these variations, specifically focusing on the differences in work‐from‐home practices by commuting time across municipalities.

The work‐from‐home data are derived from a survey conducted by Okubo (2021),^15^ ^,^ ^16^ The pre‐pandemic data represent the work‐from‐home proportion in January 2020, while the post‐pandemic data reflect the proportion in April or May 2020. These values are presented in the second and third columns of Supporting Information S1: Appendix Table B3. Before and after the onset of COVID‐19, the proportion of individuals working from home significantly varied by commuting time. In general, longer commuting times were associated with a higher tendency to work from home. The pandemic led to an increase in remote work across all commuting time categories, with the largest rise of 21% points among those with 90–120 min commutes. This trend highlights the substantial impact of COVID‐19 on work and commuting behaviors.

## 4 Results

### 4.1 Main Results

The baseline results for females and males are shown in Table 1. The first columns of Tables 1a and 1b present the ordinary least squares (OLS) estimates for females and males. However, the OLS estimates are likely to be biased because of possible confounding factors, such as psychological and environmental traits. For example, if poorer mental health is associated with a higher propensity to stay at home and higher suicide risk, the OLS estimate will be upward biased. To address this concern, we employ the shift‐share IV.^17^ The first stage results of Equation (5) and IV results of Equation (4) are presented in Columns (2) and (3), respectively. The first‐stage results show a clear and strong association between the difference in the stay‐at‐home variable and difference in the shift‐share IV. In addition, the effective F‐statistic exceeds the threshold value at the conventional level (Pflueger and Wang 2015), thus rejecting the null hypothesis of a weak IV. The IV coefficients for both females and males remain positive. However, the estimates for women are no longer statistically significant. Column (4) presents the reduced‐form results from Equation (7). The estimates of the shift–share IV are positive for both females and males, indicating that municipalities more exposed to the COVID‐19 shock experienced greater increases in suicide rates, although the female estimate is not statistically significant.

**TABLE 1:** Effect of staying at home on suicide.

	OLS	1st Stage	IV	Reduced form
	(1)	(2)	(3)	(4)
(a) Female				
ΔStayHome	1.109		0.827	
	(0.432)		(0.554)	
	[0.010]		[0.136]	
ΔShiftShare		1.620		1.339
		(0.037)		(0.901)
		[0.000]		[0.137]
Effective F statistic		1964.183		
τ = 10%		23.109		
Observations	1241	1241	1241	1241
(b) Male				
ΔStayHome	0.745		1.058	
	(0.335)		(0.424)	
	[0.026]		[0.012]	
ΔShiftShare		1.620		1.714
		(0.037)		(0.687)
		[0.000]		[0.013]
Effective F statistic		1964.183		
τ = 10%		23.109		
Observations	1241	1241	1241	1241

*Note:* We estimate the first‐order time difference as in Equation (4). In the regression results in Columns (1), (3), and (4), the outcome variable is ln(1+suicidecases). Further, we convert the coefficient to the semi‐elasticity of suicide using the equation specified in footnote 3. In the regression results in Column (2), the outcome variable is the stay‐at‐home variable. Heteroskedasticity robust standard errors are in parentheses. *p*‐values are in brackets. The effective F‐statistics are those developed by Montiel Olea and Pflueger (2013).

Importantly, the data collected during the pandemic show heterogeneity in the incidence of suicide. As discussed in Section 1, an increase in suicide during COVID‐19 was not uniform but was more pronounced in some demographic groups, especially young people. To examine the heterogeneity in the effect, we conducted a subsample analysis by age group. We consider the following age groups: those under 20, in their 20s; in their 30s; in their 40s; in their 50s; and aged 60 years or older.

Table 2 reports the IV and reduced‐form estimates separately for females and males, revealing pronounced age‐specific heterogeneity in both groups. For females, the stay‐home effect is sharply concentrated among those under 20, being twice as large effect for them compared to other age groups, and is statistically significant at conventional levels. Specifically, the magnitude of the increase is approximately 5%, associated with a 1% point increase in the staying‐at‐home variable for females under 20. Meanwhile, the corresponding value is 1%–2% for all other age groups, with the oldest group showing the least pronounced increase. We analyze the magnitude of this effect to discuss the degree of attribution of staying at home to an increase in suicide incidence by conducting a counterfactual analysis in Section 4.4. The results from the reduced‐form analysis are qualitatively consistent with the IV results. For males, as reported in Table 2b, the IV coefficients are positive across all age brackets but are generally smaller and imprecisely estimated; only the 40–49 cohort shows a statistically significant increase of approximately 1.5%, which is smaller than the effect observed among females under 20 years.

**TABLE 2:** IV estimates by age group.

	(1)	(2)	(3)	(4)	(5)	(6)
	Under 20	20–29	30–39	40–49	50–59	Over 60
(a) Female						
Panel A: IV						
ΔStayHome	4.823	1.492	1.760	1.338	1.743	1.213
	(2.026)	(1.189)	(1.291)	(0.989)	(1.048)	(0.772)
	[0.017]	[0.210]	[0.173]	[0.176]	[0.096]	[0.116]
Panel B: Reduced form						
ΔShiftShare	7.811	2.416	2.851	2.167	2.822	1.965
	(3.288)	(1.930)	(2.096)	(1.603)	(1.701)	(1.253)
	[0.018]	[0.211]	[0.174]	[0.176]	[0.097]	[0.117]
Observations	1241	1241	1241	1241	1241	1241
(b) Male						
Panel A: IV						
ΔStayHome	1.251	0.692	0.768	1.447	1.000	0.255
	(1.525)	(0.891)	(0.804)	(0.747)	(0.826)	(0.680)
	[0.412]	[0.437]	[0.340]	[0.053]	[0.226]	[0.708]
Panel B: Reduced form						
ΔShiftShare	3.048	1.641	2.051	3.395	2.372	0.479
	(3.716)	(2.114)	(2.150)	(1.757)	(1.963)	(1.281)
	[0.412]	[0.437]	[0.340]	[0.053]	[0.227]	[0.709]
Observations	1241	1241	1241	1241	1241	1241

*Note:* Heteroskedasticity‐robust standard errors are in parentheses; *p*‐values are in brackets. The dependent variable is ln(1+suicidecases). The coefficients are converted to semi‐elasticities following Footnote 3. Subtables (a) and (b) present the results for female and male age groups, respectively. In each subtable, Panel A shows the IV estimates from Equation (4) and Panel B shows the estimates from Equation (7).

Notably, while the outcome is age‐specific, both the stay‐at‐home measure and its shift–share instrument are aggregated across all demographic groups. Consequently, the estimates for young females not only capture the effect of their own confinement but also the spillovers from the confinement of others. For instance, family relationships and intergenerational social interactions, rather than solely through their own confinement, may have an influence.

### 4.2 Estimation Validity

To confirm the validity of the findings, we conduct robustness checks, focusing on two age‐gender groups, females under 20 years and males aged 40–49 years, which show statistically significant impacts. By following the framework outlined by Goldsmith‐Pinkham et al. (2020), we examine the necessary conditions required for the shift‐share IV estimator to be unbiased.

### 4.3 Group‐Specific Time Trend

In the differential exposure design, a principal concern is the presence of unobserved variations in characteristics among municipalities which were classified into the treatment and control categories. The observed changes in suicide rates among young females may not exclusively stem from the differences in commuting time distributions. Other factors, such as the degree of urbanization and nature of local industries, may also play a significant role. If these elements drive the suicide trend after the pandemic started, the observed correlation between post‐COVID‐19 stay‐at‐home increases and suicide rates, as indicated by the regression analyses, may not necessarily reflect a causal relationship.

To establish a causal relationship, the time trends specific to municipal attributes, termed group‐specific trends, must be controlled. The structural models incorporating these differential trends can be formulated as follows:

(9) $$\Delta \;\log\left(1+Suicide_{m}\right)=\delta +\beta \Delta StayHome_{m}+\sum_{s=1}^{S}\phi_{s}W_{sm}+\Delta \nu_{m}$$

Here, $W_{m}=\left(W_{1m},\text{…},W_{Sm}\right)$ is a set of municipal characteristics. $\phi_{s}$ represents a common time trend for groups sharing the characteristic s, which serves as a factor loading in a linear factor model for time‐series analysis. The equation is derived by taking the difference in the following expression between t=1 (the pandemic period) and t=0 (the pre‐pandemic period):

$$\log\left(1+Suicide_{mt}\right)=\alpha +\delta \times t+\beta StayHome_{mt}+\sum_{s=1}^{S}\phi_{s}\left(t\times W_{\mathrm{smt}}\right)+\nu_{mt}.$$

The baseline Equation (4) incorporates an unobserved error component, expressed as $\Delta \varepsilon_{m}=\sum_{s=1}^{S}\phi_{s}W_{sm}+\Delta \nu_{m}$ of Equation (9). If the specific time trends tied to each group influence suicide incidence within municipalities, this could compromise the exclusion restriction given by Equation (8), as $z_{km}$ and $W_{sm}$ may be correlated for some k and s. Such a scenario can generate an omitted variable bias when estimating the causal effects of home confinement on suicide using the baseline model.

Acknowledging the potential influence of group‐specific time trends, Table 3 presents the additional regression analyses, incorporating urban‐ and industry‐specific trends as factor loadings in estimating Equation (9). The results in Column (1) serve as the baseline in Table 2, which shows the estimates without considering group‐specific time trends for reference. The findings in Columns (2) and (3) indicate that including these trends does not significantly alter the estimated impact of stay‐at‐home orders on suicide. Specifically, when controlling for urbanization with a dummy variable denoting whether a municipality is a city, the effect of home confinement on suicide incidence remains statistically significant at the 5% level. Conversely, when industries are categorized into primary, secondary, and tertiary sectors, and we account for the varying ratios of industrial composition within municipalities, the estimated impact of home confinement on suicide is positive but not statistically significant. Nonetheless, a small change in these estimates from the baseline after incorporating several controls indicates that group‐specific trends do not drive our previous findings.

**TABLE 3:** The Effect of stay‐at‐home on suicides: Robustness check.

	(1)	(2)	(3)	(4)	(5)	(6)	(7)	(8)	(9)	(10)
(a) Female under 20										
ΔStayHome	4.823	4.532	4.192	6.333	4.760	5.163	5.029	5.010	5.319	5.260
	(2.026)	(2.012)	(2.750)	(3.189)	(2.126)	(2.224)	(2.302)	(2.302)	(2.206)	(2.478)
	[0.017]	[0.024]	[0.127]	[0.047]	[0.025]	[0.020]	[0.029]	[0.037]	[0.016]	[0.034]
(b) Male 40–49										
ΔStayHome	1.447	1.422	1.603	0.841	0.968	1.572	1.143	2.009	1.377	1.081
	(0.747)	(0.752)	(1.079)	(1.139)	(0.852)	(0.818)	(0.893)	(0.985)	(0.712)	(0.905)
	[0.053]	[0.059]	[0.137]	[0.460]	[0.256]	[0.054]	[0.201]	[0.041]	[0.053]	[0.232]
Group specific trends										
City		x		x						
Industry			x	x						
Others				x						
Local labor market										
Unemployment rate					x		x			
Active job opening ratio						x	x			
COVID cases and deaths								x		
Functional form	Log‐linear	Log‐linear	Log‐linear	Log‐linear	Log‐linear	Log‐linear	Log‐linear	Log‐linear	Ihs‐linear	Log‐linear
IV	SS	SS	SS	SS	SS	SS	SS	SS	SS	SOE
Effective F statistics	1964.183	1990.049	1164.216	836.720	1677.856	1654.951	1535.249	1274.997	1964.183	1164.216
τ=10%	23.109	23.109	23.109	23.109	23.109	23.109	23.109	23.109	23.109	23.109
Observations	1241	1241	1241	1241	1241	1239	1239	1241	1241	1241

*Note:* Heteroskedasticity‐robust standard errors are in parentheses. *p*‐values are in brackets. In Columns (1)–(8) and (10), the outcome variable is ln(1+suicidecases). The outcome variable is transformed in Column (9) with the inverse hyperbolic sine function. In group‐specific trends, City is a dummy variable indicating municipal classification as a city; Industry represents the shares of the primary, secondary, and tertiary sectors; and Others encompass variables such as the youth, self‐employment, labor force participation, and single‐person household rates, all as outlined in Supporting Information S1: Appendix Table B6. In Column (10), the IV is constructed using the geographical variation in the state of emergency orders. SS denotes the shift–share IV, and SOE denotes the share of days under the state of emergency used as an IV.

In addition to differences in urbanization and industry types, variations in other municipality attributes may also affect the prevalence of suicide, influenced by group‐specific commuting patterns. However, accounting for factor loadings across all municipality attributes and controlling for them in regression analyses is a daunting task because numerous potential attributes could drive these group‐specific trends. Instead, we focus on the factors that strongly correlate with the share type with the highest Rotemberg weight, encompassing 60–90‐min commuters. Rotemberg weights highlight the contribution of each type of instrument to the estimate, as further discussed in this subsection. Given that Rotemberg weights emphasize the instruments most susceptible to endogeneity in the estimated parameters, as explored in Goldsmith‐Pinkham et al. (2020), these attributes can introduce notable estimation bias via group‐specific trends, which requires thorough investigation. Supporting Information S1: Appendix Table B6 details the regression analysis results of the share of 60–90‐min commuters against community characteristics in the pre‐COVID‐19 period. It reveals significant correlations between this commuter share and key municipality attributes, such as age composition, employment patterns, and the prevalence of single‐person households.^18^

A factor‐loading model, represented by Equation (9), is estimated to control for time trends in the municipality characteristics highly correlated with the share of 60–90‐min commuters, and the city and industry dummies found in Supporting Information S1: Appendix Table B6. For females under 20, the results in Column (4) of Table 3 reveal that the estimates remain positive and are statistically distinct from zero. Therefore, even after adjusting for group‐specific trends, home confinement has a positive causal effect on suicide among young women. By contrast, for males aged 40–49 years, the coefficient in Column (4) decreases and no longer statistically significant. These findings suggest that the observed increase in suicides in this group is unlikely to have been driven by home confinement.

### 4.4 Time‐Varying Labor Market Conditions, Pandemic Severity, and Other Functional Forms

Another concern is the unobservable changes in labor market conditions and pandemic intensity. Here, we conduct an analysis considering these factors. The details about the data used here are in Supporting Information S1: Appendix C.

First, the results after controlling for labor market conditions are presented in Columns (5)–(7) of Table 3. In Japan, research indicates a correlation between worsening labor market conditions, particularly unemployment, and an increase in suicide rates (Chen et al. 2009; Chen et al. 2015). Thus, a causal relationship may exist linking stay‐at‐home behaviors, which could exacerbate downturns in the regional economy, to worsened local labor demand conditions, and consequently, increased suicide. To check whether this path exists, we include the unemployment rate and job opening‐to‐applicant ratio for each municipality in our analysis. The results for females under 20 years indicate that the estimated parameter of the stay‐at‐home variable remains positive and statistically significant even after controlling for labor market conditions. Notably, when compared to the baseline estimates without controls for labor market conditions, the magnitude of this effect is slightly greater. This suggests that prima facie, the impact of home confinement on the suicide of women under 20 years is a direct effect rather than one mediated through declining labor market conditions.^19^

For males aged 40–49 years, controlling for unemployment rates reduces the magnitude of the estimated impact and the coefficients are no longer statistically significant. This contrasts with the findings for females under 20 years. Thus, a part of the observed effect may have been mediated by labor market conditions during the pandemic, which is consistent with earlier studies on the link between economic conditions and male suicide (Chen et al. 2009; Chen et al. 2015).

Second, we control for pandemic severity, as measured by COVID‐19 case counts and related deaths. This is important, as some observed impacts may reflect fear of infection, which should be separated from the impact of staying at home on mental health. However, including these controls does not qualitatively alter the findings for either group.

To enhance robustness, we conduct further analysis on our model's functional form. Initially, we replace the $\log\left(1+Suicide_{mt}\right)$ transformation with an inverse hyperbolic sine transformation for the dependent variable, defined as $\arcsin\left(Suicide_{mt}\right)=\log\left(Suicide_{mt}+\sqrt{1+Suicide_{mt}^{2}}\right)$. The results in Column (9) of Table 3 continue to demonstrate a statistically significant effect of the stay‐at‐home variable on suicide.

Finally, we perform an additional analysis using an alternative instrumental variable based on the geographical variation in state‐of‐emergency orders, rather than the shift–share design.^20^ We rely on the shift–share IV for the main analysis because state‐of‐emergency orders may be endogenous and could violate the exclusion restriction. The results, reported in Column (10), show a comparable magnitude of the estimated effect, with statistical significance observed only among females under age 20.

### 4.5 Rotemberg Decomposition

We use the *Rotemberg decomposition*, as introduced in Section 2.3, to evaluate the robustness of our estimated outcomes. This technique decomposes the estimated value of the structural equation, $\hat{\beta }$, which relies on the shift‐share instrument $ShiftShare_{mt}$, into a weighted sum. Specifically, $\hat{\beta }$ consolidates the individual estimates ${\hat{\beta }}_{k}$ that originate from a distinct IV $z_{mk}$ for k=0,…,5, resulting in $\hat{\beta }=\sum_{k}{\hat{\alpha }}_{k}{\hat{\beta }}_{k}$. The Rotemberg weights ${\hat{\alpha }}_{k}$ play a pivotal role in identifying which instruments might be prone to misspecification. For shift‐share instruments, these weights identify the shares that potentially introduce bias, particularly when the exclusion restriction, as detailed in Equation (8), is invalid. Considering the previous robustness check results, henceforth, the discussion is primarily on females under the age of 20.

The Rotemberg decomposition results for females under 20 are presented in Panel A of Table 4. A full range of diagnostic statistics is reported in Supporting Information S1: Appendix B7. Using the shares of individuals with varying commuting times in municipalities during the pre‐COVID‐19 period as IVs, we find that the instrument with the highest Rotemberg weight is associated with the share of individuals with 60–90 of commuting time. This is followed by the share of individuals commuting for 30–60 min.^21^

**TABLE 4:** Rotemberg diagnosis.

	${\hat{\alpha }}_{k}$	${\hat{\beta }}_{k}$	${\hat{F}}_{k}$	95% CI
Panel A: Shares				
Commuting time (60,90] mins	0.659	6.820	1681.096	[2.448, 11.213]
Commuting time (30,60] mins	0.551	2.415	722.220	[‐2.618, 7.456]
Commuting time (90,120] mins	0.185	4.451	439.269	[−1.651, 10.416]
Commuting time > 120 min	0.003	4.014	26.637	[−12.010, 20.775]
Commuting time (0,30] mins	−0.397	4.620	1807.293	[0.626, 8.595]

	60–90 min	30–60 min	60–90 min 30–60 min	All
Panel B: IV estimates				
ΔStayHome	6.820	2.415	5.173	4.505
	(2.246)	(2.570)	(2.040)	(2.002)
	[0.002]	[0.347]	[0.011]	[0.024]
Effective F statistics	1681.096	722.220	881.521	11.026
τ = 10%	23.109	23.109	11.618	22.854
Over ID test			2.964	7.131
			[0.085]	[0.129]
Observations	1241	1241	1241	1241

*Note:* Heteroskedasticity‐robust standard errors are in parentheses. *p*‐values are in brackets. Panel A presents the shift‐share diagnostics as recommended by Goldsmith‐Pinkham et al. (2020). The computation is based on a Stata package developed by the authors. Panel A reports four key metrics for the Rotemberg decomposition: Rotemberg weights ${\hat{\alpha }}_{k}$, the just‐identified coefficients ${\hat{\beta }}_{k}$, and the first‐stage F‐statistics for the just‐identified instruments ${\hat{F}}_{k}$ for each commuter type k. The 95% confidence intervals are robust to weak instruments, as calculated using the method proposed by Chernozhukov and Hansen (2008), spanning from −10.00 to 5.00 in increments of 0.01. Panel B presents the estimated coefficients for the impact of stay‐at‐home on suicide among females under 20 years old, associated with the highest and second‐highest Rotemberg weight shares (60–90 and 30–60 min), employing a single instrument approach (as depicted in the first and second columns). It further presents the coefficients estimated using these two shares as instruments (in the third column) and those obtained by employing all commuter shares as multiple instruments (in the fourth column). Additionally, we include the first‐stage F statistic and, where applicable, the *p*‐value for the Sargan overidentification test to assess the validity of the IVs.

We employ the shares with the highest and second‐highest Rotemberg weights as individual and combined instruments to investigate the impact of the stay‐at‐home variable on suicide among females under 20 years of age. The findings, outlined in Table 4 Panel B, demonstrate that the estimated effects are in line with the shift‐share instrument findings. The results of the Montiel–Olea and Pflueger tests (Montiel Olea and Pflueger 2013) show that the hypothesis of weak IVs is rejected when we use the shares of individuals with 60‐90‐ and 30–60‐min commutes both as a single instrument and in combination. Conversely, they are weak when all commuting time brackets are employed separately as multiple instruments. Additionally, to address potential misspecifications when multiple IVs are used, we conduct Hansen's J‐test for overidentification. According to the F‐statistic and *p*‐value in Table 4, the exogeneity of the share instrument $z_{mk}$ is not rejected at the 5% significance level.

For males aged 40–49, the results are shown in Supporting Information S1: Appendix Table B8. The estimates using the shares with the highest and second‐highest Rotemberg weights as instruments are consistent with those from the shift‐share instrument. Similar to females under 20, when each commuting time bracket is used as a separate instrument, the IV becomes weak.

### 4.6 Parallel Pre‐Trend

A potential threat to our identification strategy is the presence of pre‐trends. Although we have demonstrated visual evidence of parallel pre‐trends in Section 2.2, relying solely on this evidence is inadequate for statistical analysis. Therefore, we implement additional checks for parallel pre‐trends, as suggested by Goldsmith‐Pinkham et al. (2020).

**FIGURE 4:**
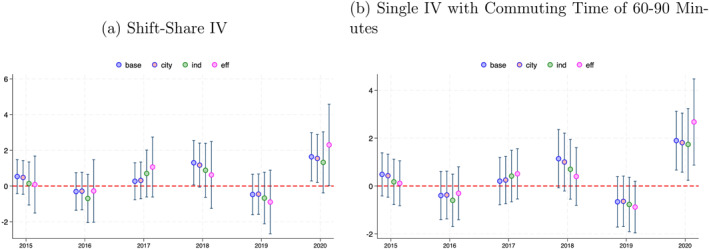
Pre‐trend test: females under 20. (a) Shift‐share IV. (b) Single IV with commuting time of 60–90 minutes. The dot shows the estimated semi‐elasticities of suicide with respect to the exposure variable (a) commuting time instrumental variable (IV) and (b) commuting time 60–90 IV for females under 20 years of age with their 95% confidence intervals for the post‐ (2020) and pre‐COVID‐19 periods (2015, 2016, 2017, 2018, and 2019). The semi‐elasticity is calculated based on the estimated coefficient $\gamma_{\ell }$ of Equation (10). In each panel, we show the estimates from the baseline specification along with three alternative specifications presented as robustness checks in Table 3. The “base” specification includes no controls; “city” adds a dummy variable indicating whether a municipality is classified as a city; “ind” controls for industrial composition; and “eff” includes other municipal attributes correlated with the commuter share. The details are provided in the main text under the section Group‐specific time trend.

Specifically, we perform a regression analysis in which our primary focus is to examine the predictive power of these instruments for changes in suicidal behavior in the *pre‐COVID‐19 period*. Figure 4 plots the coefficient of the IV for the following reduced‐form regression for females under 20 years:

(10) $$\Delta \;\ln\left(1+Suicide_{m\ell }\right)=\delta^{r}+\gamma_{\ell }Z_{m}+\Delta \varepsilon_{m}^{r},$$

where $\Delta \;\ln\left(1+Suicide_{m\ell }\right)\equiv \ln\left(1+Suicide_{m\ell +1}\right)-\ln\left(1+Suicide_{m\ell }\right)$ represents the l‐th order difference in the logarithm of the number of suicides of females under 20 in the *lead direction*. The IV $Z_{m}$ can take one of two forms, $\Delta ShiftShare_{m}$ or the highest Rotemberg weight share $z_{m3}$, which is the share of commuters with a 60–90‐min commute. To facilitate comparison, we plot the baseline estimates at ℓ=0 for 2020, representing the coefficients from the baseline reduced‐form regression as outlined in Equation (7). Here, ℓ=0 denotes 2020 as the reference point. Further, we extend our analysis to ℓ=−5, which corresponds to 2015. To ensure robustness, we estimate the main specification without the covariates alongside the three alternative specifications used in the robustness checks.^22^ We use the same months as in the main analysis from April to March.

Across all four specifications in both panels of Figure 4, we find no evidence of a systematic pre‐pandemic trend. The coefficient for 2018 is not statistically significant at the 5% level in all but one case and its magnitude is small. Thus, the 2018 estimate likely reflects a random variation rather than a meaningful pre‐trend. These findings support our identifying assumption that the share‐based IVs are uncorrelated with the pre‐pandemic trends in suicidal behavior among females under 20 years.

Supporting Information S1: Appendix Figure B1 presents the same analysis for males aged 40–49 years. While the pre COVID coefficients for males aged 40‐49 show no clear violation of the parallel trends assumption, the 2020 coefficient is imprecise and becomes insignificant once more controls are included. This instability suggests that any rise in suicides for this group is more likely to be driven by other factors than by stay‐at‐home behavior.

### 4.7 Specification Curve Analysis

A growing literature has discussed the credibility and reproducibility of research in economics and other fields (Christensen and Miguel 2018). To demonstrate the sensitivity of the results to model specifications and other decisions regarding the research design, we conduct a specification curve analysis (Simonsohn et al. 2020). This approach visualizes the magnitude of the estimated effect across various specifications and presents the effects sorted by magnitude. Thus, the specification curve analysis acts as both an extension and formalization of traditional robustness checks.

Effectively, we explore the different model specifications by considering various factors such as the presence or absence of group‐specific trends, local labor market conditions, and pandemic severity, which may correlate with commuter share, as shown in Supporting Information S1: Appendix Table B6. In addition, we consider the choice of IVs (utilizing a shift‐share variable as one IV or the segment with the highest Rotemberg weight as a single IV), time periods (three period choices: including March 2020 and 2021; including March 2021 but not March 2020; and excluding March 2020 and 2021), and functional forms (log‐linear or inverse hyperbolic sine transformations). The total number of specifications is 6144 and the first‐stage regression's F value exceeds 100 for all formulations.

**FIGURE 5:**
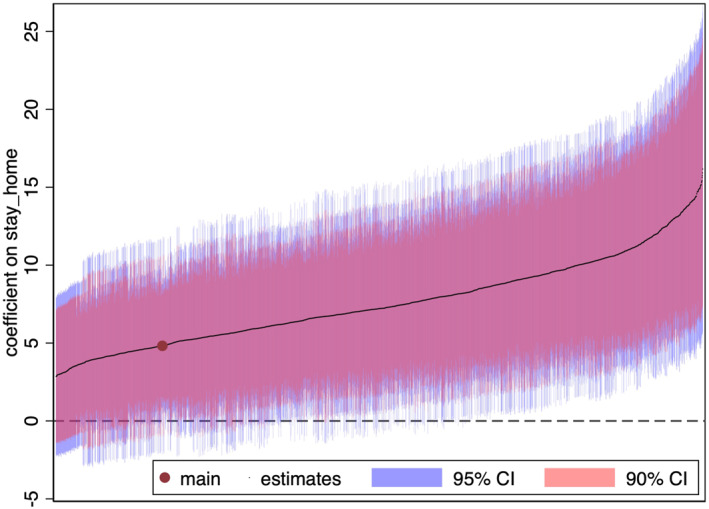
Specification curve analysis. The figure shows the specification curve analysis for females under age 20. Each dot shows the semi‐elasticity of suicide with respect to the stay‐at‐home variable, with different sets of covariates, instrumental variables (IV), and sample periods. The total number of specifications is 6,144, with the first‐stage F value exceeding 100 in all specifications. Specifically, 6144 is the product of 2 function forms × 2 IV choices $\times \;2^{6}$ (with and without 6 factors for time trend) $\times \;2^{3}$ (with and without 2 control variables for economic conditions and pandemic severity) × 3 period choices (including March 2020 and 2021; including March 2021 but not March 2020; and excluding March 2020 and 2021). The red dot is the estimate in our baseline specification. The pink and purple bars show the 90% and 95% confidence intervals, respectively.

Figure 5 displays the estimated parameters $\hat{\beta }$ pertinent to these models for females under 20 years arranged in order of magnitude.^23^ The red dot corresponds to our baseline estimate in Table 2. The smallest estimate is 2.857 (at the left end of the graph), while the largest is 16.775 (at the right end). In 70.02% of the specifications, the coefficient is significantly different from zero at the 5% level, which reinforces the robust causal relationship between stay‐at‐home behavior and suicide rates in females under 20.

Supporting Information S1: Appendix Figure B2 presents the results for males aged 40–49, which are less robust than those for other age‐gender groups. In 27.7% of specifications, the coefficient is statistically significant at the 5% level.

### 4.8 Counterfactual Analysis

Based on the aforementioned results, we perform a counterfactual analysis to quantify the extent to which the increase in suicide among females under 20 years can be attributed to stay‐at‐home behavior. We do not conduct the same analysis for males aged 40–49 years, given the less robust findings for that group. Using the estimated empirical model, we calculate the counterfactual number of suicide cases at the pre‐COVID‐19 stay‐at‐home level, and compare it with the predicted number of suicides with the actual post‐COVID‐19 stay‐at‐home level. Specifically, we estimate our model by using Equation (1), and obtain ${\hat{\alpha }}_{m}$, $\hat{\delta }$, and $\hat{\beta }$. Then, for each municipality m, we predict the counterfactual/actual suicide incidence using the stay‐at‐home variable in the pre‐ and post‐COVID‐19 periods using the following equation:

(Counterfactual) $$\ln\left(1+{\hat{Suicide}}_{m,counterfactual}\right)=\hat{\alpha_{m}}+\hat{\delta }\times 1+\hat{\beta }StayHome_{m,0}+\varepsilon_{m,1},$$

(Actual) $$\ln\left(1+{\hat{Suicide}}_{m,actual}\right)=\hat{\alpha_{m}}+\hat{\delta }\times 1+\hat{\beta }StayHome_{m,1}+\varepsilon_{m,1}.$$

In each equation, ${\hat{\alpha }}_{m}$, $\hat{\delta }$, $\hat{\beta }$ denote the estimates from Equation (1), while ${\hat{\text{Suicide}}}_{m,\;\text{counterfactual}}$ and ${\hat{\text{Suicide}}}_{m,\;\text{actual}}$ represent the predicted counterfactual and actual suicide cases for municipality m, respectively. In addition, t=0 denotes the pre‐pandemic period and t=1 denotes the pandemic period.

Finally, we calculate the aggregate change in suicide incidence across all municipalities and report the proportional difference from the pre‐pandemic level using the following equation:

(11) $$\Delta \hat{Suicide}=\frac{\sum_{m=1}^{M}\left({\hat{Suicide}}_{m,actual}-{\hat{Suicide}}_{m,couterfactual}\right)}{\sum_{m=1}^{M}{\hat{Suicide}}_{m,counterfactual}}.$$

where M is the number of municipalities. In this simulation, we select two model specifications that correspond to the smallest (2.857) and largest point (16.775) estimates $\hat{\beta }$ in the specification curve analysis 4.3. Therefore, we can consider the calculated numbers as lower and upper bounds of the contribution of stay‐at‐home behavior.

The total number of suicides predicted by the counter factual equation ranges from 178.5 to 190.5, while the “Actual” number of suicides ranges from 253.1 to 256.5. Thus, our simulation reveals that approximately 34.7%–41.8% of national suicides were caused by home confinement. Thus, the effect of stay‐at‐home behavior was substantial.^24^

## 5 Discussion

Our IV estimates indicate that the increase in suicides among young females was attributable to home confinement across various model specifications with different IV choices.

Although not every suicide can be linked to a diagnosis of mental illness, suicide is closely associated with mental health conditions (Yeh et al. 2019). COVID‐19 mental health research has identified young females as a specific high‐risk group, consistent with our findings. Review papers on the effects of the pandemic on mental health have identified risk factors for distress, including female gender identity, adolescent age, young adult age, and student status (Xiong et al. 2020; Penninx et al. 2022). Furthermore, using a large‐scale, nationally representative survey in the UK, a study reported that the prevalence of psychiatric disorders and loneliness was greater among females and young people than among the remaining the population, suggesting that young females are more vulnerable to the pandemic than others (Li and Wang 2020). While the COVID‐19 pandemic affected almost the entire population, these studies suggest that the mental effects of lifestyle changes induced by the pandemic were especially detrimental to this particular population. Our results are consistent with these findings, indicating that physical disconnection from society is a key pathway for the adverse effects of suicide among young females.

Our results are not necessarily inconsistent with U.S. research showing that increased suicide is associated with a return to in‐class schooling (as opposed to school closure) (Hansen et al. 2022). We do not aim to examine the effect of schooling mode on mental health. Rather, we focus on the effect of home confinement, which includes a wider aspect of lifestyle changes, including relationships with family members and the opportunity to meet friends outside school. The variation we exploit to identify the impact of home confinement differs from that used to identify the impact of a change in the schooling mode.

Using a regression discontinuity design based on an age cutoff, a closely related study demonstrated that the lockdown worsened the mental health of elderly people (Altindag et al. 2022). Although the RD design sharply identifies the effect of lockdown policies, the nature of the design does not permit an analysis of how the effect differs by age. Meanwhile, our shift‐share IV estimate indicates that, in the context of Japan, heterogeneity is important. Further, the effect on disconnection from society is more serious among young people in terms of suicide, which is the worst consequence of mental disorders.

Although we were unable to determine why staying at home would affect only young females, several possible mechanisms exist via which home confinement affects suicide through mental health deterioration. Social disconnection through home confinement increases the risk of mental illness morbidity through the feelings of isolation and loneliness (Loades et al. 2020). In addition, in the COVID‐19 context, people tended to stay at home across the population, including adults. Therefore, some of the observed effects among young people may reflect family‐related issues such as domestic violence or abuse, as reported in other countries (Leslie and Wilson 2020; Arenas‐Arroyo et al. 2021; Berniell and Facchini 2021; Hsu and Henke 2021). Furthermore, staying at home may hinder access to treatment and care in mental health services. All these factors may be associated with the observed increased risk of suicide. An analysis of the causes of Japanese youth suicides during the pandemic using time‐series data revealed periodic increases attributed to family related and social concerns, whereas suicides linked to mental illness particular increased as the pandemic progressed into the later months of 2020 (Goto et al. 2022).

Early public health responses against the COVID‐19 pandemic included social distancing measures, such as lockdowns and stay‐at‐home orders. These measures could also be policy options for future infectious‐disease pandemics, especially before pharmaceutical interventions become available. However, the adverse effects of these pandemic responses on the mental health of children and adolescents have been reported in the context of H1N1 influenza and SARS (Sprang and Silman 2013). Our results further show that the effect was also devastating in the case of the COVID‐19 pandemic, demonstrating that collective social distancing policies impose high social costs on both individuals and society in the most severe case, as lives are especially lost among young females. Policy‐making should be built upon the awareness of such risks, with countermeasures for high‐risk populations as a part of an effort to enhance pandemic preparedness.

## Funding

This research is supported by JSPS Kakenhi (Grant ID: 20K01731, 21H00672, 22H04911) as well as the Joint Usage/Research Center, Institute of Economic Research, Hitotsubashi University (Grant ID: IERPK2411).

## Conflicts of Interest

The authors declare no conflicts of interest.

## Permission to Reproduce Material From Other Sources

The authors have nothing to report.

## Supporting information


Supporting Information S1

## Data Availability

The data that support the findings of this study are openly available in CovidSuicidePublic at https://github.com/haru8603/CovidSuicidePublic.

## Endnotes

^1^
According to a government report, the economic cost of suicide was 1.9 trillion yen, converted at the average 2010 exchange rate of 87.7 JPY/USD.

^2^
The COVID‐19 policy details are found in Supporting Information S1: Appendix A.1.

^3^
In the following tables and figures, we report the semi‐elasticity of suicide with respect to mobility, calculated from the estimated coefficient $\hat{\beta }$ based on the following equation: $\hat{\beta }\cdot \left(1+Suicide\right)/Suicide$. We can multiply the numbers in the tables by 100 to obtain a percentage change in suicide.

^4^
We adopt this time frame because the number of COVID‐19 cases rapidly increased at the end of March, while the government declared the first state of emergency on April 7, as discussed in Supporting Information S1: Section A.1. We do not consider the period after April 2021, when vaccines became available and people subsequently started returning to normal life. We do examine the sensitivity of our estimation results to the choice of periods through the specification curve analysis in Section 4.3.

^5^
See Nunn and Qian (2014) and Kearney and Levine (2015) for similar identification strategies under the differential exposure design.

^6^
The number includes 772 non‐ordinance‐designated cities, 75 wards of ordinance‐designated cities, 23 wards of Tokyo, 743 towns, and 189 villages. Ordinance‐designated cities are major Japanese cities with over 500,000 residents that have administrative powers similar to those of prefectures and are divided into wards.

^7^
Specifically, population ranges from 178 in Aogashima Village to 903,346 in Setagaya Ward, while land area spans from 3.47 square kilometers in Funahashi Village to 2177 square kilometers in Takayama City, according to the 2020 census.

^8^
The National Police Agency (NPA) gathers the underlying data, which the MHLW then publishes; see MHLW (2021). In Japan, only doctors can issue death certificates. The Medical Practitioners Law requires them to report unusual deaths to the NPA within 24 h. The NPA then conducts comprehensive investigations, including physiological tests, reviews of suicide notes and emails, family interviews, and document assessments to determine the cause of death.

^9^
For other demographic characteristics, if there are two or fewer suicides in a municipality, the number is masked for confidentiality reasons. Hence, we are unable to use these data for the analysis.

^10^
Agoop is funded by SoftBank, one of Japan's largest cell phone companies, and possesses the GPS information of SoftBank cell phone users.

^11^
Population count data provided by Agoop's Papilio service are structured to provide data by prefecture, by municipality, and at specific locations. For municipalities and specific locations, the data can distinguish whether individuals are residents of the municipality or come from different municipalities. To define our municipality‐specific stay‐at‐home measure, we use nighttime and daytime population counts of residents only, and exclude individuals coming from other municipalities.

^12^
SafeGraph foot‐traffic data are widely used in U.S. mobility studies to gauge stay‐at‐home behavior. The dataset reports each area's *time spent at home*; for instance, Yan et al. (2021) used the median value, whereas Hsu and Henke (2021) calculated the share of devices that remain at home all day at the county level. Such information is unavailable in the Agoop data.

^13^
Google's Mobility Index provides information on how the time people spend at home has changed since the start of the pandemic compared to pre‐pandemic levels. While the Google Mobility Index captures changes in time spent at home, it is only available at the prefecture level, with a mere 47 variations.

^14^
The publicly available dataset aggregates statistics at the municipality level.

^15^
Okubo (2021) conducted a nationwide survey of workers in December 2020 to understand changes in work style, lifestyle, and attitudes caused by the COVID‐19 pandemic. The total number of responses were 10,523. The work‐from‐home data for each commuting time used in our analysis are based on Table 1–17–1 in Okubo (2021), and are aggregated from more detailed time brackets.

^16^
As illustrated in Section 2.2, non‐commuting residents are assumed to always work from home $\left(g_{00}=g_{01}=1\right)$.

^17^
As a robustness check to verify that our IV findings do not hinge on the specific commuting‐time categories, we re‐estimate the model after merging the two longest commuting‐time categories; the results are reported in Supporting Information S1: Appendix Tables B4 and B5. The estimates remain qualitatively identical to those in Table 1 for both females and males.

^18^
Employment patterns include labor force rates, and self‐employment rate.

^19^
These regression results should be interpreted with caution. The local job opening‐to‐applicant ratio, which is potentially influenced by our treatment variable, staying at home, exhibits a “bad control” problem as described by Angrist and Pischke (2008). This can introduce bias into our estimates. Supporting Information S1: Appendix D provides a detailed discussion of the potential bias, explained using a *Directed Acyclic Graph (DAG)*.

^20^
In this analysis, we replace $\Delta ShiftShare_{m}$ with $\Delta SOEshare_{p}$ in Equation (5), where $\Delta SOEshare_{p}$ denotes the share of days under the state of emergency in prefecture p, to which municipality m belongs, between April 2020 and March 2021.

^21^
According to the shares perspective by Goldsmith‐Pinkham et al. (2020), our shift‐share instrument is equivalent to using the shares of individuals with varying commuting times as instruments. Thus, the exogeneity assumption should be interpreted within the context of these shares.

^22^
The three specifications correspond to Columns (2), (3) and (4), respectively, in Table 3.

^23^
To illustrate which specifications correspond to each estimate, further details are provided in Supporting Information S1: Appendix Figures B3 and B4.

^24^
According to our data, the actual number of suicides among females under 20 years of age during the pre‐COVID‐19 period was 218.
