# Supplementary material for: The Effect of Voluntary Staying at Home on Japanese Female Suicide During the COVID‐19 Pandemic
Source: Health Econ. 2026 Jan 11;35(5):742–58. doi: 10.1002/hec.70078 (PMC13039786; doi:10.1002/hec.70078)

Appendix for

"The Effect of Voluntary Staying at Home on Japanese Female Suicide during the COVID-19 Pandemic"

# A Additional information

## A.1 Background

**COVID-19 policies:** The first COVID-19 cases in Japan were reported on January 16, 2020, with limited increase in the number of cases until the end of March. The earliest measure taken was to order the closure of schools at the end of February. In response, most schools decided to close until the semester ended at the end of March.

The first action taken by the government, which affected all of Japanese society, was the declaration of the state of emergency. On April 7, 2020, the first state of emergency was declared in seven prefectures, including Tokyo[25]. Nine days later, the state of emergency was extended to all Japanese prefectures. The first declaration of the state of emergency decreased the number of people leaving their homes by 8.5% (Watanabe and Yabu, 2021). The state of emergency was gradually lifted depending on the pandemic situation[26]. Even after the state of emergency was lifted, mobility did not substantially recover because of various government interventions for social distancing. These interventions included encouraging working or studying from home, and the early closure of restaurants and bars. In addition to the effects of government interventions, people's behavior was affected by new social norms (Takahashi and Tanaka, 2021). Consequently, staying at home continued until March 2021, as discussed in Section 3.

**Trend in suicide:** In Japan, suicide cases reached their highest records in 1998 after the economic crisis and remained high until 2009. However, because of the government's efforts to prevent suicide, the number of cases decreased until the pandemic began. In 2020, the suicide rate increased for the first time since 2010. Figure A1 shows the suicide rates between April 2019 and March 2021. For females, the suicide rate was approximately 8 per 10,000 people as of June 2020; however, the trend then began rising, reaching the highest level of more than 13 per 10,000 people and remaining at a higher level in the first half of 2021. For males, the relationship with COVID-19 was less clear than that for females.

[25]These prefectures include Tokyo, Kanagawa, Chiba, and Saitama, as well as Osaka, Hyogo, and Fukuoka.
[26]Decisions on declaring and terminating the state of emergency were mainly made with the following three factors: the number of cases per population, medical surges, and polymerase chain reaction testing volume for COVID-19.

Figure A1: Suicide incidence per 100,000 population, April 2019 – March 2021

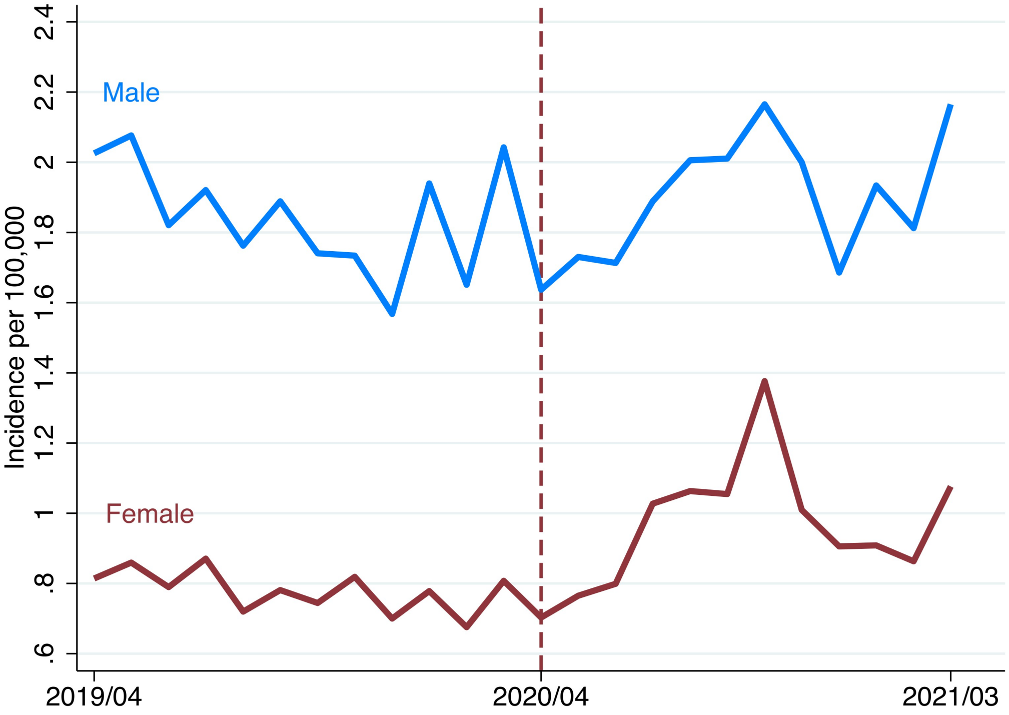


*Notes:* The data show the suicide incidence per 100,000 people in Japan between April 2019 and March 2021. A vertical dotted line marks April 2020, dividing the analysis into two periods: Pre- (prior to April 2020) and Post-COVID-19 (from April 2020 onwards).

## A.2 Foot traffic data

The stay-at-home variable significantly various across different types of municipalities, reflecting distinct patterns of resident mobility. For instance, business districts experience a large influx of nonresidents and some resident outflow during the day, resulting in lower values. Meanwhile, suburban areas show a significant daytime reduction in the resident population due to commuting, leading to higher values. Detailed explanations and observable fluctuations in the stay-at-home variable are in Appendix Figure A2, which depicts the distinctions between a business district and suburban residential area.

Figure A2: Foot Traffic Data: Examples

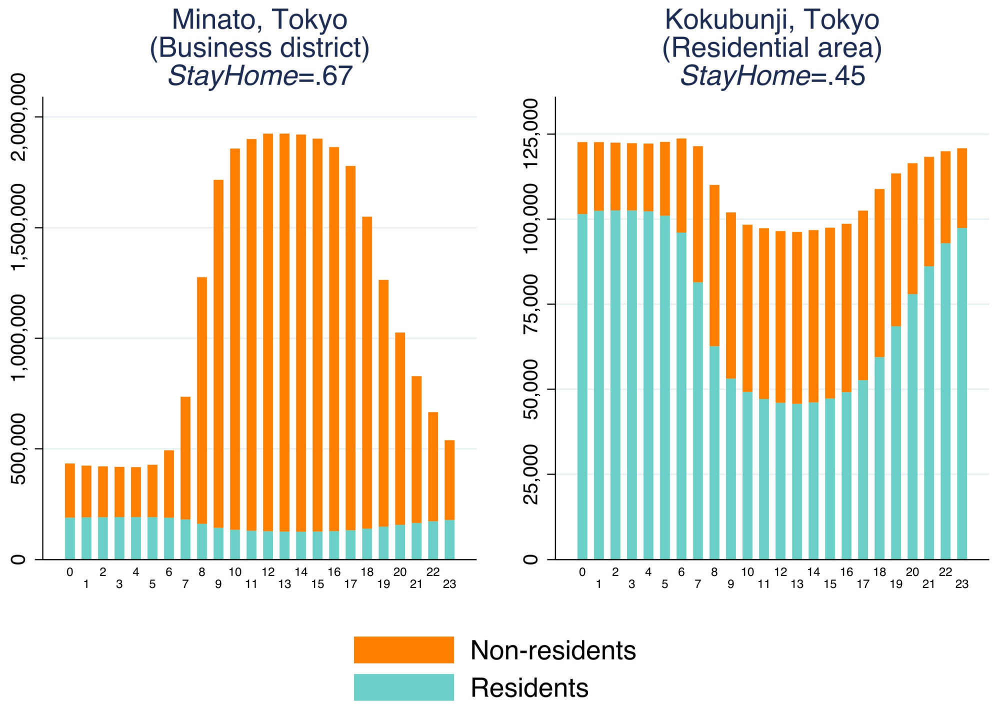


*Notes:* Light green and orange show resident and nonresident population counts, respectively, for each hour. The data show the average population in each hour in April 2019. Minato Ward, Tokyo, is an example of a business district, while Kokubunji City, Tokyo, is an example of a residential district. The stay-at-home rate is 0.67 for Minato Ward and 0.45 for Kokubunji City. A greater stay-at-home value indicates a higher tendency to stay in the municipality.

## A.3 Correlation with the Google mobility index

To confirm the reliability of our stay-at-home variable, we examine the correlation with the Google Mobility Index. This index offered timely information on individual travel during the COVID-19 pandemic. The daily mobility data are publicly available as "COVID-19: Community Mobility Report." Japanese data are available at the prefecture level starting in February 2020. The data are available as a change compared to the median value of a reference day, which is the same day of the week during the pre-pandemic period spanning from January 3 to February 6. Among the six location categories, we focus on residence as our measure of stay-at-home behavior. The Google Mobility Index shows the percentage change in the time spent in the residential area from the pre-pandemic period. We use the monthly average of the daily data of the prefecture-level Google Mobility Index between February 2020 and December 2021, and obtain the correlation coefficient with the difference in the monthly stay-at-home variable between January 2020 and each month at the prefecture level using a total of 1081 (=23 months 47 prefectures) data points. The correlation between our stay-at-home variable and the Google mobility index is 0.74.

Next, we closely examine the correlation in the time-series direction. Figure A3 in the Appendix shows the time-series plot of the stay-at-home variable and Google Mobility Index for residences between February 2020 and December 2021. Our stay-at-home variable closely follows the Google mobility index of residence throughout the analysis period.

Furthermore, we explore this association regionally. Figure A4 in the Appendix shows the association between the stay-at-home variable and Google Mobility Index of residence for each month from February 2020 to March 2021. In the first two panels, which show the association in the pre-pandemic period, there is almost no regional variation in either variable. However, diversity emerges starting in April 2020. In all sample periods, we see a clear positive correlation, confirming that our measure shows stay-at-home behavior at the individual level.

Figure A3: Time Series Association between the Stay-at-Home Variable and the Google Mobility Index

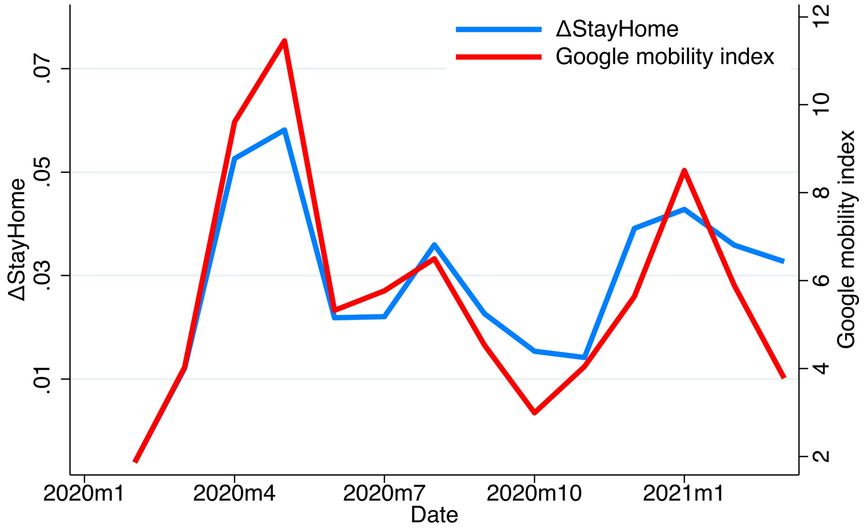


*Notes*: The figure shows the plot of the national average of $\Delta StayHome$ (blue) and Google Mobility Index (red). $\Delta StayHome$ shows the change from January 2020 to be consistent with the definition of the Google Mobility Index.

Figure A4: Regional Association between the Stay-at-Home Variable and Google Mobility Index

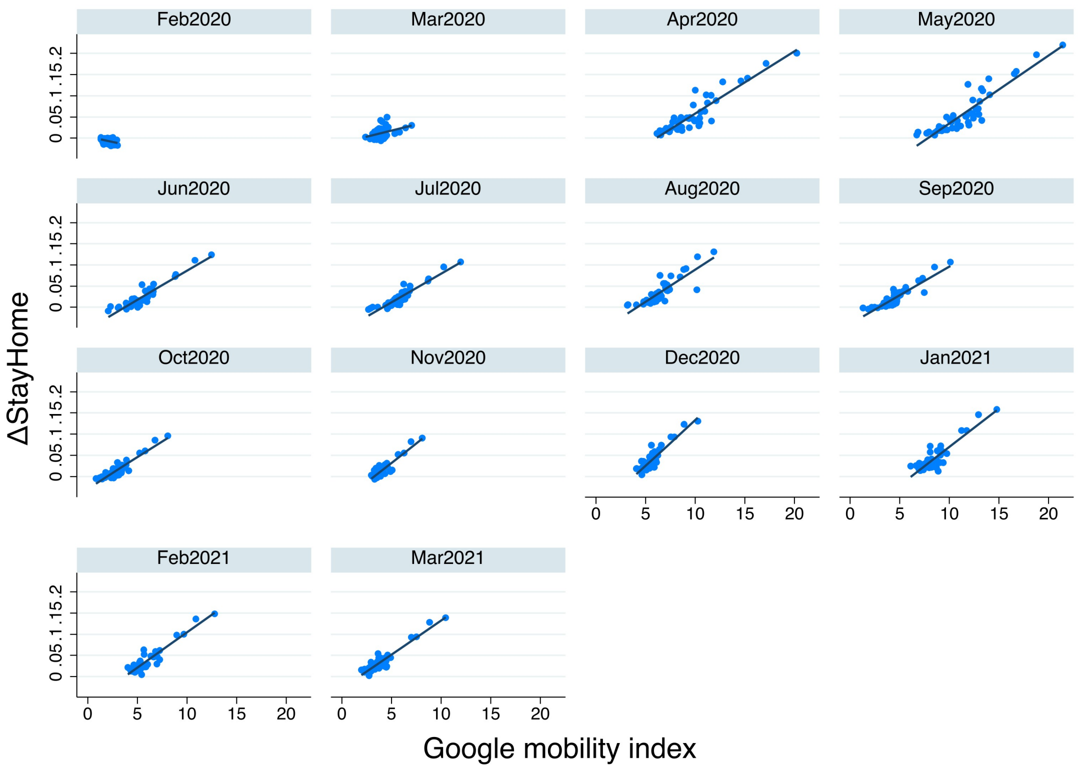


*Notes*: Each panel shows the scatterplot of the prefecture average of $\Delta StayHome$ and its linear fit. $StayHome$ shows the weighted average of the municipality-level data to obtain the prefecture average. $\Delta StayHome$ shows the change from January 2020 to be consistent with the definition of the Google Mobility Index.

# B Additional figures and tables

Figure B1: Pre-trend Test: Males 40-49

(a) Shift-share IV

(b) Single IV with Commuting Time of 60-90 Minutes

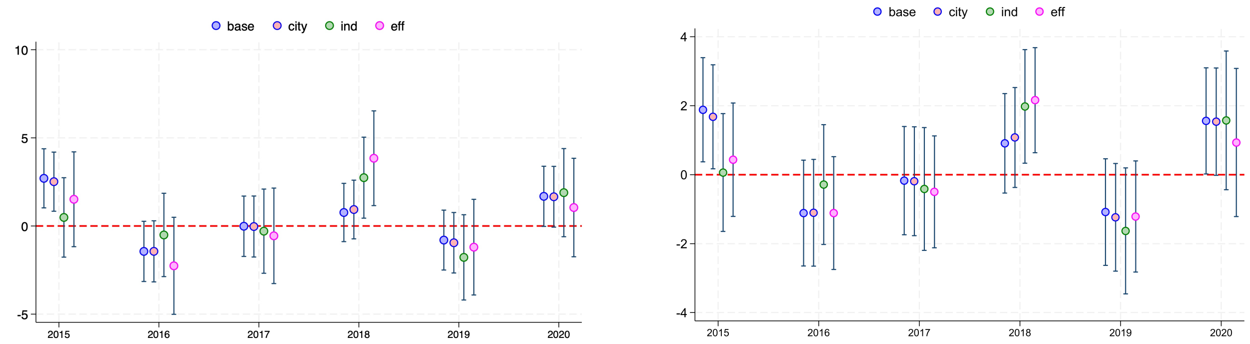


*Notes*: The dot shows the estimated semi-elasticities of suicide with respect to the exposure variable (a) commuting time IV and (b) commuting time 60-90 IV for females under 20 years of age with their 95% confidence intervals for the post- (2020) and pre-COVID-19 periods (2015, 2016, 2017, 2018, 2019). The semi-elasticity is calculated based on the estimated coefficient $\gamma_\ell$ of Equation (10). In each panel, we show estimates from the baseline specification along with three alternative specifications presented as robustness checks in Table 3. The "base" specification includes no controls; "city" adds a dummy variable indicating whether a municipality is classified as a city; "ind" controls for industrial composition; and "eff" includes other municipal attributes correlated with the commuter share. The details are provided in the main text under the section Group-specific Time Trend.

Figure B2: Specification Curve Analysis for Males aged 40—49 years

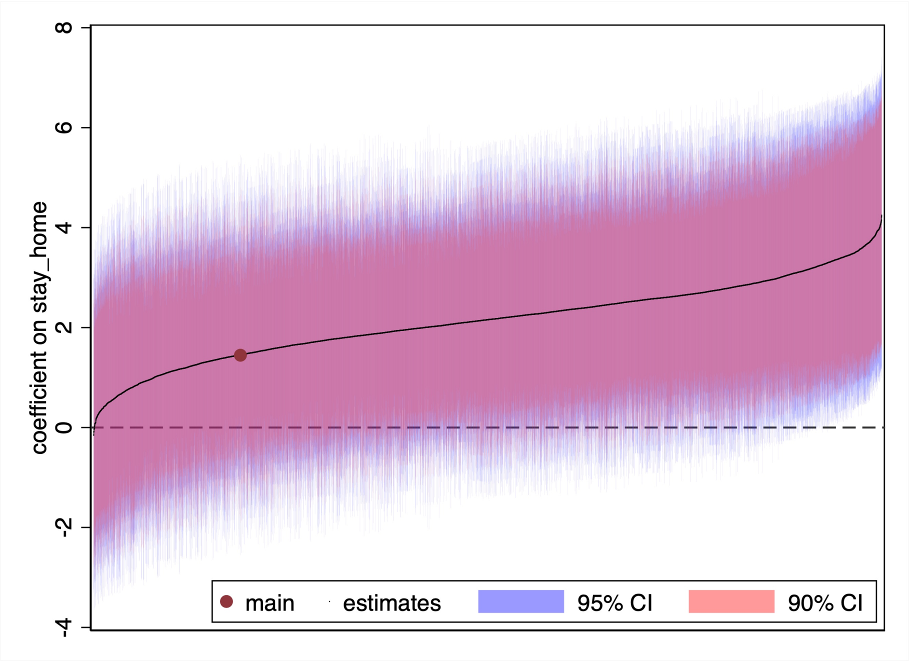


*Notes*: The figure shows the specification curve analysis for males under age 20. Each dot shows the semi-elasticity of suicide with respect to the stay-at-home variable, with different sets of covariates, instrumental variables (IVs), and sample periods. The total number of specifications is 6,144, with the first-stage F value exceeding 100 in all specifications. Specifically, 6,144 is the product of 2 function forms $\times$ 2 IV choices $\times$ $2^6$ (with and without 6 factors for time trend) $\times$ $2^3$ (with and without 2 control variables for economic conditions and pandemic severity) $\times$ 3 period choices (including March 2020 and 2021; including March 2021 but not March 2020; and excluding March 2020 and 2021). The red dot is the estimate in our baseline specification. The pink and purple bars show the 90% and 95% confidence intervals, respectively.

Figure B3: Specification Curve Analysis: Details for Females under 20

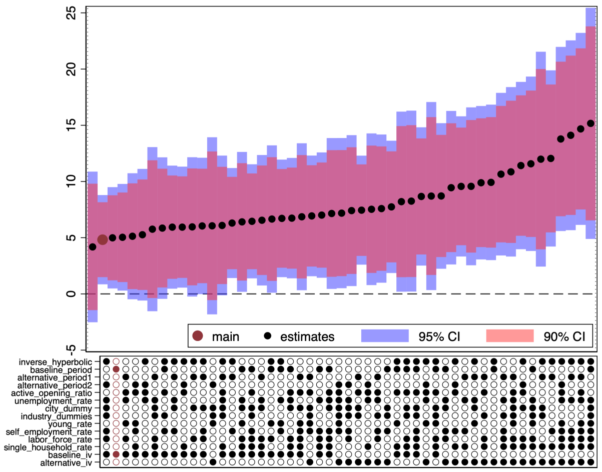


(a) Selected at random

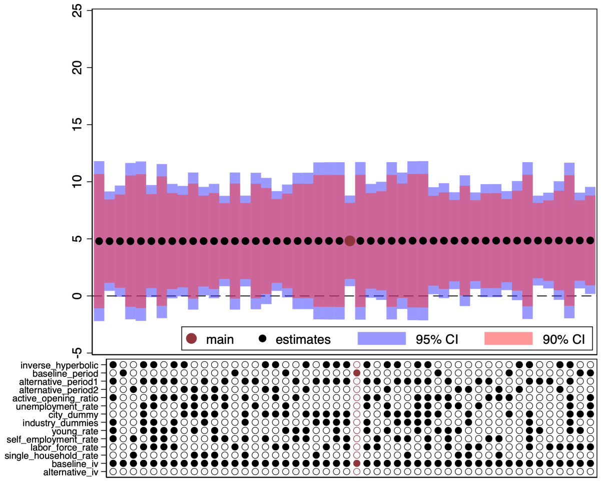


(b) Around baseline

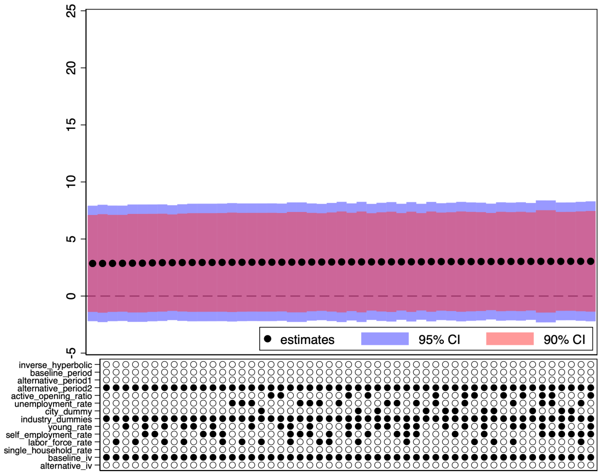


(c) Bottom 50

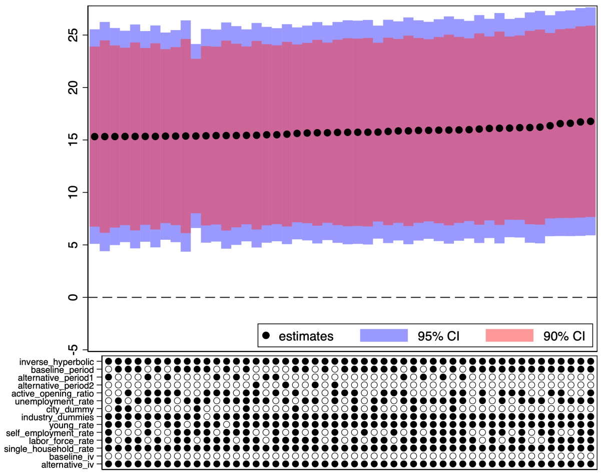


(d) Top 50

*Notes*: The figure shows the specification curve analysis for females under 20. Each dot shows the semi-elasticity of suicide with respect to the stay-at-home variable, with different sets of covariates, instrumental variables, and sample periods. The details of the specifications are found in the notes of Figure 5. The red dot marks the baseline estimate; each panel plots roughly 50 specifications—random in (a), near-baseline in (b), bottom in (c), and top in (d).

Figure B4: Specification Curve Analysis: Details for Males 40-49

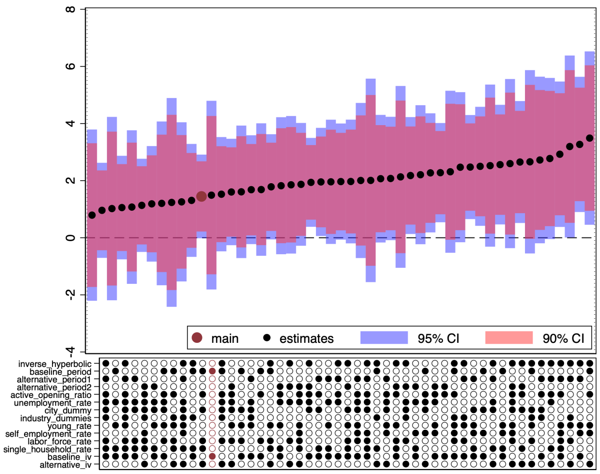


(a) Selected at random

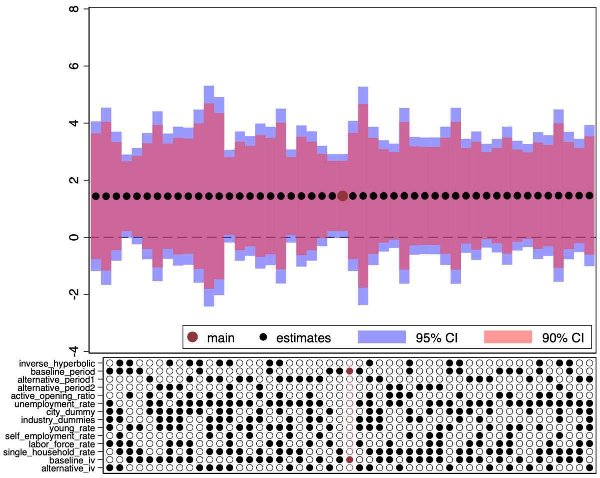


(b) Around baseline

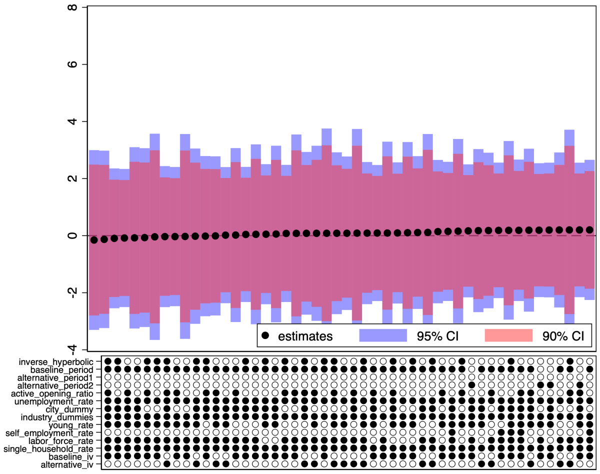


(c) Bottom 10%

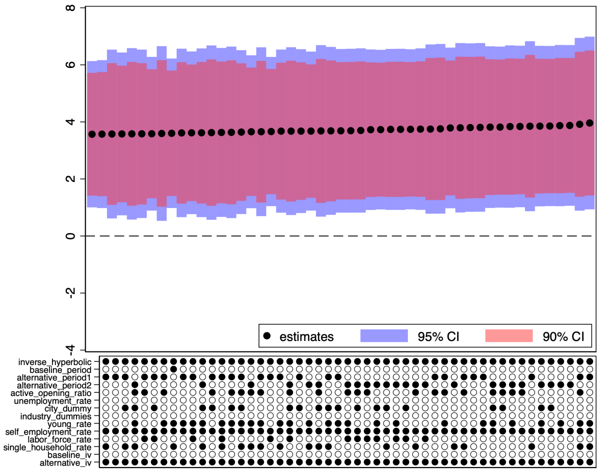


(d) Top 10%

*Notes*: The figure shows the specification curve analysis for males aged 40-49 years using the stay-at-home variable. Each dot shows the semi-elasticity of suicide with respect to the stay-at-home variable, with different sets of covariates, instrumental variables, and sample periods. The details of the specifications are found in the notes of Figure 5. The red dot marks the baseline estimate; each panel plots roughly 50 specifications—random in (a), near-baseline in (b), bottom in (c), and top in (d).

Table B1: Descriptive Statistics

| | Total | | | | Before | | After | |
|---|---|---|---|---|---|---|---|---|
| | Mean | Std. Dev. | Min | Max | Mean | Std. Dev. | Mean | Std. Dev. |
| *Panel A: Suicide and Stay-at-home variable* | | | | | | | | |
| Female suicide | 5.175 | 6.266 | 0 | 48 | 4.633 | 5.534 | 5.717 | 6.880 |
| Female suicide under 20 | 0.213 | 0.532 | 0 | 5 | 0.176 | 0.475 | 0.251 | 0.581 |
| Female suicide 20-29 | 0.612 | 1.245 | 0 | 17 | 0.507 | 1.041 | 0.716 | 1.412 |
| Female suicide 30-39 | 0.550 | 1.037 | 0 | 10 | 0.469 | 0.941 | 0.630 | 1.119 |
| Female suicide 40-49 | 0.828 | 1.325 | 0 | 11 | 0.719 | 1.171 | 0.938 | 1.455 |
| Female suicide 50-59 | 0.796 | 1.314 | 0 | 10 | 0.716 | 1.204 | 0.876 | 1.413 |
| Female suicide 60s or over | 2.175 | 2.575 | 0 | 20 | 2.044 | 2.416 | 2.305 | 2.719 |
| Male suicide | 10.667 | 11.519 | 0 | 87 | 10.529 | 11.241 | 10.804 | 11.793 |
| Male suicide under 20 | 0.359 | 0.746 | 0 | 7 | 0.330 | 0.723 | 0.388 | 0.767 |
| Male suicide 20-29 | 1.249 | 1.878 | 0 | 18 | 1.134 | 1.748 | 1.364 | 1.995 |
| Male suicide 30-39 | 1.408 | 1.964 | 0 | 17 | 1.395 | 1.900 | 1.421 | 2.027 |
| Male suicide 40-49 | 1.909 | 2.429 | 0 | 18 | 1.898 | 2.397 | 1.919 | 2.462 |
| Male suicide 50-59 | 1.849 | 2.415 | 0 | 20 | 1.878 | 2.395 | 1.820 | 2.435 |
| Male suicide 60s or over | 3.891 | 4.209 | 0 | 30 | 3.893 | 4.227 | 3.889 | 4.192 |
| StayHome | 0.670 | 0.123 | 0 | 1 | 0.640 | 0.132 | 0.701 | 0.106 |
| *Panel B: Shares, shifts, and IV* | | | | | | | | |
| Commuting ratio | 0.464 | 0.194 | 0.009 | 0.787 | 0.464 | 0.195 | 0.464 | 0.195 |
| Commuting time 0 mins | 0.017 | 0.009 | 0.000 | 0.114 | 0.017 | 0.009 | 0.017 | 0.009 |
| Commuting time (0,30] mins | 0.589 | 0.175 | 0.142 | 0.929 | 0.589 | 0.175 | 0.589 | 0.175 |
| Commuting time (30,60] mins | 0.270 | 0.100 | 0.033 | 0.610 | 0.270 | 0.100 | 0.270 | 0.100 |
| Commuting time (60,90] mins | 0.090 | 0.084 | 0.000 | 0.431 | 0.090 | 0.084 | 0.090 | 0.084 |
| Commuting time (90,120] mins | 0.025 | 0.029 | 0.000 | 0.213 | 0.025 | 0.029 | 0.025 | 0.029 |
| Commuting time > 120 mins | 0.009 | 0.008 | 0.000 | 0.086 | 0.009 | 0.008 | 0.009 | 0.008 |
| Work from home ratio (commuting time 0 mis) | 1.000 | 0.000 | 1.000 | 1.000 | 1.000 | 0.000 | 1.000 | 0.000 |
| Work from home ratio (commuting time (0,30] mins) | 0.055 | 0.025 | 0.030 | 0.080 | 0.030 | 0.000 | 0.080 | 0.000 |
| Work from home ratio (commuting time (30,60] mins) | 0.153 | 0.078 | 0.075 | 0.230 | 0.075 | 0.000 | 0.230 | 0.000 |
| Work from home ratio (commuting time (60,90] mins) | 0.200 | 0.094 | 0.106 | 0.294 | 0.106 | 0.000 | 0.294 | 0.000 |
| Work from home ratio (commuting time (90,120] mins) | 0.214 | 0.106 | 0.108 | 0.320 | 0.108 | 0.000 | 0.320 | 0.000 |
| Work from home ratio (commuting time > 120 mins) | 0.223 | 0.021 | 0.202 | 0.244 | 0.202 | 0.000 | 0.244 | 0.000 |
| Shift-share IV | 0.116 | 0.053 | 0.040 | 0.261 | 0.069 | 0.013 | 0.163 | 0.032 |
| *Panel C: Time-invariant covariates* | | | | | | | | |
| Primary industry rate | 0.060 | 0.063 | 0.000 | 0.395 | | | | |
| Secondary industry rate | 0.259 | 0.075 | 0.081 | 0.512 | | | | |
| Tertiary industry rate | 0.681 | 0.096 | 0.430 | 0.919 | | | | |
| Young rate | 0.124 | 0.019 | 0.057 | 0.207 | | | | |
| Middle rate | 0.583 | 0.043 | 0.413 | 0.748 | | | | |
| Elderly rate | 0.285 | 0.057 | 0.149 | 0.518 | | | | |
| Labor force rate | 0.569 | 0.039 | 0.387 | 0.699 | | | | |
| Self-employment rate | 0.100 | 0.032 | 0.042 | 0.246 | | | | |
| Single person household rate | 0.139 | 0.028 | 0.032 | 0.231 | | | | |
| City | 0.782 | 0.413 | 0.000 | 1.000 | | | | |
| *Panel D: Time-varying covariates* | | | | | | | | |
| Active opening ratio | 1.361 | 0.644 | 0.000 | 8.935 | 1.586 | 0.723 | 1.136 | 0.453 |
| Unemployment rate | 2.436 | 0.493 | 1.250 | 3.600 | 2.195 | 0.386 | 2.677 | 0.471 |
| Covid patients | 198.323 | 539.576 | 0 | 3,311 | 0.943 | 1.811 | 395.703 | 710.307 |
| Covid death cases | 3.085 | 4.227 | 0 | 14 | 0.000 | 0.000 | 6.171 | 4.086 |
| Observations | 2,482 | | | | 1,241 | | 1,241 | |

*Notes*: The sample period is between April 2019 and March 2021. Before COVID-19 indicates the period between April 2019 and March 2020, while after COVID-19 indicates the period between April 2020 and March 2021. The definitions of the variables are provided in the main text or in Appendix C.

## Table B2: Descriptive Statistics for Analytic versus Excluded Samples

| | Before | | | After | | |
|---|---|---|---|---|---|---|
| | Included | Excluded | Diff of Means | Included | Excluded | Diff of Means |
| *Panel A: Suicide and Stay-at-home variable* | | | | | | |
| Female suicide | 4.633 | 0.311 | -4.322 | 5.717 | 0.388 | -5.329 |
| | [5.534] | [0.589] | (0.159) | [6.880] | [0.671] | (0.197) |
| Female suicide under 20 | 0.176 | 0.008 | -0.168 | 0.251 | 0.015 | -0.235 |
| | [0.475] | [0.087] | (0.014) | [0.581] | [0.146] | (0.017) |
| Female suicide 20-29 | 0.507 | 0.017 | -0.490 | 0.716 | 0.023 | -0.693 |
| | [1.041] | [0.129] | (0.030) | [1.412] | [0.160] | (0.041) |
| Female suicide 30-39 | 0.469 | 0.017 | -0.452 | 0.630 | 0.032 | -0.598 |
| | [0.941] | [0.129] | (0.027) | [1.119] | [0.177] | (0.033) |
| Female suicide 40-49 | 0.719 | 0.032 | -0.686 | 0.938 | 0.035 | -0.903 |
| | [1.171] | [0.177] | (0.034) | [1.455] | [0.193] | (0.042) |
| Female suicide 50-59 | 0.716 | 0.046 | -0.670 | 0.876 | 0.057 | -0.819 |
| | [1.204] | [0.210] | (0.035) | [1.413] | [0.232] | (0.041) |
| Female suicide 60s or over | 2.044 | 0.191 | -1.854 | 2.305 | 0.225 | -2.081 |
| | [2.416] | [0.445] | (0.071) | [2.719] | [0.511] | (0.080) |
| Male suicide | 10.529 | 0.892 | -9.637 | 10.804 | 0.845 | -9.960 |
| | [11.241] | [1.101] | (0.322) | [11.793] | [1.080] | (0.337) |
| Male suicide under 20 | 0.330 | 0.018 | -0.312 | 0.388 | 0.015 | -0.373 |
| | [0.723] | [0.135] | (0.021) | [0.767] | [0.123] | (0.022) |
| Male suicide 20-29 | 1.134 | 0.063 | -1.071 | 1.364 | 0.049 | -1.315 |
| | [1.748] | [0.243] | (0.051) | [1.995] | [0.217] | (0.057) |
| Male suicide 30-39 | 1.395 | 0.114 | -1.281 | 1.421 | 0.097 | -1.325 |
| | [1.900] | [0.341] | (0.056) | [2.027] | [0.301] | (0.059) |
| Male suicide 40-49 | 1.898 | 0.125 | -1.774 | 1.919 | 0.118 | -1.801 |
| | [2.397] | [0.374] | (0.070) | [2.462] | [0.380] | (0.071) |
| Male suicide 50-59 | 1.878 | 0.129 | -1.749 | 1.820 | 0.145 | -1.675 |
| | [2.395] | [0.354] | (0.069) | [2.435] | [0.401] | (0.071) |
| Male suicide 60s or over | 3.893 | 0.443 | -3.450 | 3.889 | 0.420 | -3.469 |
| | [4.227] | [0.720] | (0.123) | [4.192] | [0.733] | (0.122) |
| StayHome | 0.640 | 0.693 | 0.053 | 0.701 | 0.726 | 0.025 |
| | [0.132] | [0.164] | (0.007) | [0.106] | [0.163] | (0.007) |
| *Panel B: Commuting ratio* | | | | | | |
| Commuting ratio | 0.464 | 0.356 | -0.108 | | | |
| | [0.195] | [0.201] | (0.010) | | | |
| *Panel C: Time-invariant covariates* | | | | | | |
| Primary industry rate | 0.060 | 0.184 | 0.124 | | | |
| | [0.063] | [0.114] | (0.005) | | | |
| Secondary industry rate | 0.259 | 0.240 | -0.019 | | | |
| | [0.075] | [0.091] | (0.004) | | | |
| Tertiary industry rate | 0.681 | 0.576 | -0.105 | | | |
| | [0.096] | [0.097] | (0.005) | | | |
| Young rate | 0.124 | 0.108 | -0.016 | | | |
| | [0.019] | [0.027] | (0.001) | | | |
| Middle rate | 0.583 | 0.528 | -0.055 | | | |
| | [0.043] | [0.052] | (0.002) | | | |
| Elderly rate | 0.285 | 0.362 | 0.077 | | | |
| | [0.057] | [0.072] | (0.003) | | | |
| Labor force rate | 0.569 | 0.584 | 0.015 | | | |
| | [0.039] | [0.064] | (0.003) | | | |
| Self-employment rate | 0.100 | 0.156 | 0.056 | | | |
| | [0.032] | [0.049] | (0.002) | | | |
| Single person household rate | 0.139 | 0.117 | -0.023 | | | |
| | [0.028] | [0.036] | (0.002) | | | |
| City | 0.782 | 0.000 | -0.782 | | | |
| | [0.413] | [0.000] | (0.012) | | | |
| *Panel D: Time-varying covariates* | | | | | | |
| Active opening ratio | 1.586 | 1.530 | -0.056 | 1.136 | 1.179 | 0.043 |
| | [0.723] | [0.962] | (0.043) | [0.453] | [0.616] | (0.027) |
| Unemployment rate | 2.195 | 2.211 | 0.017 | 2.677 | 2.660 | -0.017 |
| | [0.386] | [0.368] | (0.018) | [0.471] | [0.450] | (0.022) |
| Covid patients | 0.943 | 0.934 | -0.009 | 395.703 | 163.832 | -231.871 |
| | [1.811] | [2.300] | (0.104) | [710.307] | [291.374] | (23.177) |
| Covid death cases | 0.000 | 0.000 | 0.000 | 6.171 | 5.949 | -0.221 |
| | [0.000] | [0.000] | (0.000) | [4.086] | [4.920] | (0.225) |
| Observations | 1,241 | 650 | 1,891 | 1,241 | 650 | 1,891 |

*Notes*:"Include" refers to observations used in our analysis, while "Excluded" refers to those lacking commuting time data. Numbers in brackets denote standard deviations, while those in parentheses indicate standard errors. Eleven municipalities without mobility data are excluded. Hence, the sample size (1,891) is slightly smaller than the total number of municipalities in Japan (1,902). The sample period is between April 2019 and March 2021. Before COVID-19 indicates the period between April 2019 and March 2020, while after COVID-19 indicates the period between April 2020 and March 2021. The definitions of the variables are provided in the main text or Appendix C.

Table B3: Descriptive Statistics for Commuter Type

| Commuting time | k | $\bar(z)_k$ | $g_{k0}$ | $g_{k1}$ |
|---|---|---|---|---|
| 0 mins | 0 | 0.017 | 1.000 | 1.000 |
| (0, 30] mins | 1 | 0.589 | 0.030 | 0.080 |
| (30, 60] mins | 2 | 0.270 | 0.075 | 0.230 |
| (60, 90] mins | 3 | 0.090 | 0.106 | 0.294 |
| (90, 120] mins | 4 | 0.025 | 0.108 | 0.320 |
| > 120 mins | 5 | 0.009 | 0.202 | 0.244 |
| > 90 mins | 4 | 0.033 | 0.142 | 0.292 |

*Notes*: Commuter type is represented by $k = 0, 1, 2, 3, 4, 5$. $\bar{z}_k$ represents the average share of each commuter type $k$ across municipalities. In each municipality $m$, the share of each commuter type is calculated based on the 2018 Housing and Land Survey of Japan. $g_{k0}$ and $g_{k1}$ represent the proportion of those who worked from home before and after the pandemic, respectively. The data are obtained from Table 1-17-1 of Okubo (2021).

Table B4: Suicide Effects of Staying at Home: Robustness with Alternative Commuting Time Categories

| | OLS | 1st Stage | IV | Reduced form |
|---|---|---|---|---|
| | (1) | (2) | (3) | (4) |
| Δ StayHome | 1.109 | | 0.805 | |
| | (0.432) | | (0.554) | |
| | [0.010] | | [0.146] | |
| Δ ShiftShare IV | | 1.701 | | |
| | | (0.038) | | |
| | | [0.000] | | |
| Δ ShiftShare IV | | | | 1.369 |
| | | | | (0.946) |
| | | | | [0.148] |
| Effective F statistic | | 1965.789 | | |
| $\tau$=10% | | 23.109 | | |
| Observation | 1,241 | 1,241 | 1,241 | 1,241 |

| | OLS | 1st Stage | IV | Reduced form |
|---|---|---|---|---|
| | (1) | (2) | (3) | (4) |
| Δ StayHome | 0.745 | | 0.999 | |
| | (0.335) | | (0.422) | |
| | [0.026] | | [0.018] | |
| Δ ShiftShare IV | | 1.701 | | |
| | | (0.038) | | |
| | | [0.000] | | |
| Δ ShiftShare IV | | | | 1.699 |
| | | | | (0.718) |
| | | | | [0.018] |
| Effective F statistic | | 1965.789 | | |
| $\tau$=10% | | 23.109 | | |
| Observation | 1,241 | 1,241 | 1,241 | 1,241 |

*Notes*: We use an alternative commuting time categorization in which the top two commuting time categories are combined ($K = 5$). We estimate the first-order time difference as in Equation (4). In the regression results in Columns (1), (3), and (4), the outcome variable is defined as ln(1 + suicide cases) , and we convert the coefficient to the semi-elasticity of suicide by using the equation specified in footnote 3. In the regression results in Column (2), the outcome variable is the stay-at-home variable. Heteroskedasticity robust standard errors are in parentheses. P-values are in brackets. The effective F statistics are those developed by Montiel Olea and Pflueger (2013).

Table B5: Suicide Effects of Staying at Home by Age Group: Robustness with Alternative Commuting Time Categories

(a) Female

| | (1) Under 20 | (2) 20-29 | (3) 30-39 | (4) 40-49 | (5) 50-59 | (6) Over 60 |
|---|---|---|---|---|---|---|
| *Panel A: IV* | | | | | | |
| $\Delta StayHome$ | 4.837 | 1.479 | 1.812 | 1.299 | 1.727 | 1.214 |
| | (2.023) | (1.183) | (1.287) | (0.990) | (1.045) | (0.771) |
| | [0.017] | [0.211] | [0.159] | [0.189] | [0.098] | [0.115] |
| *Panel B: Reduced form* | | | | | | |
| $\Delta ShiftShare$ | 7.811 | 2.416 | 2.851 | 2.167 | 2.822 | 1.965 |
| | (3.288) | (1.930) | (2.096) | (1.603) | (1.701) | (1.253) |
| | [0.018] | [0.211] | [0.174] | [0.176] | [0.097] | [0.117] |
| Observations | 1,241 | 1,241 | 1,241 | 1,241 | 1,241 | 1,241 |

(b) Male

| | (1) Under 20 | (2) 20-29 | (3) 30-39 | (4) 40-49 | (5) 50-59 | (6) Over 60 |
|---|---|---|---|---|---|---|
| *Panel A: IV* | | | | | | |
| $\Delta StayHome$ | 1.165 | 0.679 | 0.725 | 1.355 | 0.961 | 0.258 |
| | (1.529) | (0.888) | (0.801) | (0.746) | (0.823) | (0.679) |
| | [0.446] | [0.445] | [0.366] | [0.069] | [0.243] | [0.704] |
| *Panel B: Reduced form* | | | | | | |
| $\Delta ShiftShare$ | 3.048 | 1.641 | 2.051 | 3.395 | 2.372 | 0.479 |
| | (3.716) | (2.114) | (2.150) | (1.757) | (1.963) | (1.281) |
| | [0.412] | [0.437] | [0.340] | [0.053] | [0.227] | [0.709] |
| Observations | 1,241 | 1,241 | 1,241 | 1,241 | 1,241 | 1,241 |

*Notes*: We use an alternative commuting time categorization in which the top two commuting time categories are combined ($K = 5$). Heteroskedasticity-robust standard errors are in parentheses; p-values are in brackets. The dependent variable is $\ln(1 + \text{suicide cases})$. Coefficients are converted to semielasticities following Footnote 3. Subtables (a) and (b) present the results for female and male age groups, respectively. In each subtable, Panel A shows the IV estimates from Equation (4) and Panel B shows the estimates from Equation (7).

Table B6: Correlation between the Share of 60-90-Minute Commuters and Municipality Characteristics

| | (1) | (2) | (3) | (4) |
|---|---|---|---|---|
| City | 0.096 | | 0.000 | 0.016 |
| | (0.003) | | (0.005) | (0.004) |
| | [0.000] | | [0.938] | [0.000] |
| Primary industry rate | | -0.398 | -0.398 | 0.293 |
| | | (0.031) | (0.031) | (0.241) |
| | | [0.000] | [0.000] | [0.225] |
| Secondary industry rate | | -0.114 | -0.114 | -0.037 |
| | | (0.020) | (0.020) | (0.236) |
| | | [0.000] | [0.000] | [0.875] |
| Tertiary industry rate | | 0.211 | 0.211 | 0.434 |
| | | (0.008) | (0.009) | (0.226) |
| | | [0.000] | [0.000] | [0.055] |
| Young rate (under 15) | | | | -1.786 |
| | | | | (0.211) |
| | | | | [0.000] |
| Middle rate (between 15-64) | | | | 0.125 |
| | | | | (0.187) |
| | | | | [0.503] |
| Elderly rate (above 64) | | | | -0.267 |
| | | | | (0.181) |
| | | | | [0.139] |
| Employment rate | | | | 0.103 |
| | | | | (0.127) |
| | | | | [0.418] |
| Employee rate | | | | -0.010 |
| | | | | (0.098) |
| | | | | [0.919] |
| Self-employment rate | | | | -0.388 |
| | | | | (0.197) |
| | | | | [0.049] |
| Labor force rate | | | | -0.618 |
| | | | | (0.077) |
| | | | | [0.000] |
| Single person household rate | | | | 2.190 |
| | | | | (0.099) |
| | | | | [0.000] |
| Adj.R-Squared | 0.470 | 0.666 | 0.666 | 0.784 |
| Observations | 1,241 | 1,241 | 1,241 | 1,241 |

*Notes*: Heteroskedasticity-robust standard errors are in parentheses. P-values are in brackets. The dependent variable is the share of commuters whose commuting time ranges from 60 to 90 minutes (highest Rotemberg weight share). All variables are derived from the 2015 census data. The definitions of the variables are provided in the main text or Appendix C.

Table B7: Rotemberg Decomposition Diagnostics for Females under 20: Details

| | Sum | Mean | Share | | |
|---|---|---|---|---|---|
| *Panel A. Negative and positive weights* | | | | | |
| $\hat{\alpha}_k \leq 0$ | -0.397 | -0.397 | 0.221 | | |
| $\hat{\alpha}_k > 0$ | 1.397 | 0.349 | 0.779 | | |
| | $\hat{\alpha}_k$ | $\Delta g_k$ | $\hat{\beta}_k$ | $\hat{F}_k$ | Var($z_k$) |
| *Panel B. Correlations* | | | | | |
| $\hat{\alpha}_k$ | 1.000 | | | | |
| $\Delta g_k$ | 0.702 | 1.000 | | | |
| $\hat{\beta}_k$ | 0.230 | 0.508 | 1.000 | | |
| $\hat{F}_k$ | 0.058 | 0.311 | 0.680 | 1.000 | |
| Var($z_k$) | 0.765 | 0.608 | 0.370 | 0.609 | 1.000 |
| | $\hat{\alpha}_k$ | $\Delta g_k$ | $\hat{\beta}_k$ | $\hat{F}_k$ | 95 % C.I. |
| *Panel C. Share Statistics* | | | | | |
| Commuting time (0,30] mins | -0.397 | 0.050 | 4.620 | 1807.293 | [0.626, 8.595] |
| Commuting time (30,60] mins | 0.551 | 0.155 | 2.415 | 722.220 | [-2.618, 7.456] |
| Commuting time (60,90] mins | 0.659 | 0.188 | 6.820 | 1681.096 | [2.448, 11.213] |
| Commuting time (90,120] mins | 0.185 | 0.212 | 4.451 | 439.269 | [-1.651, 10.416] |
| Commuting time > 120 mins | 0.003 | 0.042 | 4.014 | 26.637 | [-12.010, 20.775] |
| | $\alpha$ weighted sum | share of overall $\beta$ | Mean | | |
| *Panel D: $\hat{\beta}_k$ for positive and negative weights* | | | | | |
| Negative | -1.835 | -0.381 | 4.620 | | |
| Positve | 6.658 | 1.381 | 4.425 | | |

*Notes*: The table presents the shift-share diagnostics as recommended by Goldsmith-Pinkham et al. (2020) for females under 20. The statistics are computed using a Stata package developed by the authors. Panel A delineates the aggregate, average, and distribution of negative and positive Rotemberg weights, $\hat{\alpha}_k$, across different commuter types $k$. Panel B elucidates the pairwise correlations among four key metrics for Rotemberg decomposition—Rotemberg weights $\hat{\alpha}_k$, the first difference in telework ratio $\Delta g_k$, the just-identified coefficients $\hat{\beta}_k$, and the first-stage F statistics for the just-identified instruments $\hat{F}_k$—and variance across municipalities for each commuter type $k$, denoted as $Var(z_k)$. Panel C shows the key statistics for each commuting time ratio, including the 95% confidence intervals robust to weak instruments, as calculated using the method proposed by Chernozhukov and Hansen (2008), spanning from $-10$ to 5 in increments of 0.01. Lastly, Panel D provides insights into the variability of the just identified coefficients $\hat{\beta}_k$ in relation to the positive and negative Rotemberg weights for each commuter type $k$.

Table B8: Rotemberg Decomposition Diagnostics for Males Aged 40-49

| | $\hat{\alpha}_k$ | $\hat{\beta}_k$ | $\hat{F}_k$ | 95 perc. C.I. |
|---|---|---|---|---|
| *Panel A: Shares* | | | | |
| Commuting time (60,90] mins | 0.659 | 1.637 | 1681.096 | [0.030, 3.246] |
| Commuting time (30,60] mins | 0.551 | 0.775 | 722.220 | [-1.036, 2.591] |
| Commuting time (90,120] mins | 0.185 | 2.541 | 439.269 | [0.427, 4.693] |
| Commuting time > 120 mins | 0.003 | -2.147 | 26.637 | [-11.459, 5.196] |
| Commuting time (0,30] mins | -0.397 | 1.311 | 1807.293 | [-0.152, 2.773] |
| | 60-90 min | 30-60 min | 60-90 min 30-60 min | All |
| *Panel B: IV estimates* | | | | |
| $\Delta StayHome$ | 1.637 | 0.775 | 1.303 | 1.332 |
| | (0.822) | (0.925) | (0.745) | (0.744) |
| | [0.046] | [0.402] | [0.080] | [0.073] |
| Effective F statistics | 1681.096 | 722.220 | 881.521 | 4.266 |
| $\tau$=10% | 23.109 | 23.109 | 11.618 | 23.010 |
| Over ID test | | | 0.923 | 6.506 |
| | | | [0.337] | [0.164] |
| Observations | 1,241 | 1,241 | 1,241 | 1,241 |

*Notes*: Heteroskedasticity-robust standard errors are in parentheses. P-values are in brackets. Panel A presents the shift-share diagnostics as recommended by Goldsmith-Pinkham et al. (2020) for males aged $40 - 49$ years. The computation is based on a Stata package developed by the authors. Panel A reports four key metrics for Rotemberg decomposition: Rotemberg weights $\hat{\alpha}_k$, the just-identified coefficients $\hat{\beta}_k$, and the first-stage F statistics for the just-identified instruments $\hat{F}_k$ for each commuter type $k$. The 95% confidence intervals are robust to weak instruments, as calculated using the method proposed by Chernozhukov and Hansen (2008) spanning from $-10.00$ to 5.00 in increments of 0.01. Panel B presents the estimated coefficients for the impact of stay-at-home on suicide among males in their 40s, associated with the highest and second-highest Rotemberg weight shares (60-90 and 30-60 minutes), employing a single instrument approach (as depicted in the first and second columns). It further presents the coefficients estimated using these two shares as instruments (in the third column) and those obtained by employing all commuter shares as multiple instruments (in the fourth column). Additionally, we include the first-stage F statistic and, where applicable, the p-value for the Sargan overidentification test to assess the validity of the IVs used.

# C Control Variables

The data sources for the control variables used in the robustness check are as follows:

**Local labor market:** We used two variables, the active job-opening ratio at the municipality level and prefecture-level unemployment rate, to control for local labor market conditions. The active job-opening ratio is frequently used to measure macroeconomic conditions at the local level. It is the ratio of active job openings to the number of active applicants. The Ministry of Health, Labour and Welfare (MHLW) provides monthly data specific to the level of the public employment security office on request. On average, one public employment security office covers only a few municipalities. We used the same number of active job-opening ratios for municipalities covered by the same public employment security office. Some municipalities were divided into several districts covered by different public employment security offices. In this case, we used the municipality's average active job-opening ratio of the public employment security offices. The active job-opening ratio was calculated by dividing the number of job openings by the number of job seekers in each public employment security office. We used the average active job opening ratio for the before- and during-COVID-19 periods.

We also included unemployment rates on a prefectural basis. Based on the *Labor Force Survey* conducted and reported quarterly by the Ministry of Internal Affairs and Communications at the national level in Japan, estimates of the prefecture-quarterly unemployment rate can be obtained using a time-series model. As only quarterly prefecture data were available, the same numbers were used for the same quarter for all municipalities in the same prefecture. We included the average unemployment rate in the quarterly data before and during the COVID-19 pandemic.

**Industry composition and city dummies:** To control for time trends, we included several variables to indicate the industry composition of the municipality and a city dummy.

The variables to indicate the industry composition show the share of workers in the 20 industries based on the Japan Standard Industry Classification (https://www.soumu.go.jp/english/dgpp_ss/seido/sangyo/san13-3.htm), which includes the following industry categories: agriculture and forestry; fisheries; mining and quarrying of stone and gravel; construction; manufacturing; electricity, gas, heat, and water; information and communications; transport and postal activities; wholesale and retail trade; finance and insurance; real estate and goods rental and leasing; scientific

research, professional and technical services; accommodations, eating and drinking services; living-related and personal services and amusement services; education, learning support; medical, health care and welfare; compound services; services, N.E.C.; government, except elsewhere classified; and industries unable to classify. We used the 2015 census data for the pre-pandemic period and the 2020 data for the pandemic period because the census survey was conducted in October of that year.

For a city dummy, which was used to control for urban areas, we created binary variables which equaled one if a municipality is a city and zero otherwise.

**COVID-19 cases and deaths:** We included the number of identified COVID-19 cases and deaths to control for local epidemics. We calculated the number of cases per 100 thousand population using public sources on COVID-19 patients and deaths.

Patient information is publicly available on the websites of each prefecture; however, the type of information regarding patient residence varies among prefectures. In certain prefectures, patient residence is disclosed at the municipality level. Meanwhile, in others, it is reported at the health center or regional level, which encompasses an area equal to or larger than a municipality. Moreover, in some instances, the reported units have changed in some prefectures. Our approach aims to maximize the utilization of information derived from the data reported by the prefectural government while ensuring consistency within municipalities over time. Accordingly, we aggregated the number of COVID-19 patients based on the municipality level for 34 prefectures, health center district level for 11 prefectures, and regional level for 2 prefectures. For some patients, residence information was missing. Hence, we excluded these patients when constructing the variable.

Data on the number of deaths from COVID-19 were obtained from the MHLW. MHLW compiles the data published by each prefecture (https://covid19.mhlw.go.jp). These data are available at the prefectural level. According to the MHLW, a COVID-19 death is defined as "the number of deceased individuals who were confirmed positive for COVID-19 based on reports under the Infectious Diseases Control Law." For both data, we used the total number of COVID-19 cases and deaths before and during the COVID-19 pandemic.

**Other variables:** Some specifications included socioeconomic and demographic information at the municipality level. Demographic information included the age composition of three age groups: under 15, 15–64, and 65 years or older. We also included labor force participation, self-employment,

and single-person household rates. The labor force participation rate is the sum of the number of people who are working and who are unemployed but seeking jobs divided by the total population aged 15 years or older. The self-employment rate is defined as follows: The numerator is the sum of those who own their business, including homeworkers and those with and without employees, and the denominator is the number of workers. The single-person household rate is the share of households consisting of a single person in of the total number of households. These variables were obtained from the 2015 Census Survey of the Statistical Bureau.

**Commuting ratio:** In the empirical model section, we established a canonical model with two types as an illustration. The commuting ratio for each municipality was obtained from the 2015 Census Survey by the Statistics Bureau. It is the sum of the number of residents working at home, and number of residents working or studying within their own municipality divided by the total number of students and workers aged 15 years or older.

# D DAG: Bad Control

Angrist and Pischke (2008) describe variables that could be impacted (post-determined) by the treatment as "bad controls," advising against their inclusion in a regression model even if their inclusion might alter the regression coefficients. Conversely, they define "good controls" as variables presumably established at the point when the regressor of interest was determined (pre-determined).

In this study's context, the treatment variable, stay-at-home behavior, is likely to affect local labor market dynamics, including unemployment rates. This aspect classifies the local labor market condition as a post-determined variable. This suggests that it might serve as a "bad control" in the regression analysis estimating the causal effect of stay-at-home behavior on suicide rates among young women.

We employ causal diagrams to distinguish when post-determined variables affected by the treatment can be considered "good" or "bad." Using directed acyclic graphs (DAGs), we provide visual summaries of causal relations in various typical scenarios. When including labor market-related variables in the regression, these summaries can provide a biased estimate of the causal effect of staying at home on young women's suicidal behaviors.

Graphically, the potential causal paths under various assumptions are illustrated through the diagrams. We represent the outcome of interest, the number of female suicides, by $Y$. The endogenous stay-at-home variable is denoted by $S$ and the shift-share IV is denoted by $Z$. If we represent labor market conditions by $M$, this variable influenced by stay-at-home $S$, and thus, affects the outcome $Y$. This influence is depicted through the path $S \rightarrow M \rightarrow Y$ so that $M$ serves as a mediator. In Figure D1a, a confounder $U$ is assumed to affect both the outcome $Y$ and endogenous variable $S$. The IV $Z$ is assumed to only affect $S$ (i.e., exclusion restriction) and $S$ influences $Y$ through $M$. The direct effect of $S$ on $Y$ when $M$ is controlled is the average change in $Y$ when $S$ is altered exogenously by one unit, known as the controlled direct effect as per Pearl (2009). To estimate the controlled direct effect using the IV $S$, we need to control $M$ to close the backdoor path $Z \rightarrow S \rightarrow M \rightarrow Y$. Hence, in this scenario, $M$ is a "good control" and should be included in the regression analysis to estimate the direct effect of $S$ on $Y$.

Conversely, Figure D1b shows a situation where the confounder $U$ not only affects $Y$ and $S$ but also $M$. Here, controlling $M$ would open a colliding path $Z \rightarrow S \rightarrow M \leftarrow U \rightarrow Y$, making it impossible to identify the direct effect of $S$ on $Y$ using $Z$ as an IV. Therefore, $M$ is a "bad control".

Additionally, Figures D1c and D1d illustrate cases in which another confounder $V$ affects both the mediator $M$ and outcome $Y$. In Figure D1c, although the confounder $U$ does not affect the mediator $M$, controlling $M$ opens the colliding path $Z \rightarrow S \rightarrow M \leftarrow V \rightarrow Y$, making $M$ a "bad control". Similarly, in Figure D1d, where the confounder $U$ affects the mediator $M$, controlling $M$ opens both paths $Z \rightarrow S \rightarrow M \leftarrow U \rightarrow Y$ and $Z \rightarrow S \rightarrow M \leftarrow V \rightarrow Y$, reinforcing $M$ as a "bad control." Therefore, in these scenarios, $M$ should not be included in the regression analysis with $Z$ as the IV.

In summary, in the situation depicted in Figure D1a, the local labor market condition $M$ is a "good control" and helps in making an unbiased estimate of the direct effect. However, in the other scenarios shown from Figure D1b to Figure D1d, $M$ becomes a "bad control" and should not be included.

Figure D1: DAG: Good or Bad Controls

(a) One confounder with $M$ unconfounded by $U$

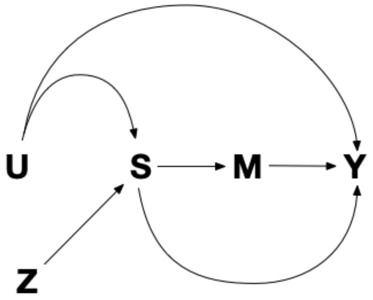


(b) One confounder with $M$ confounded by $U$

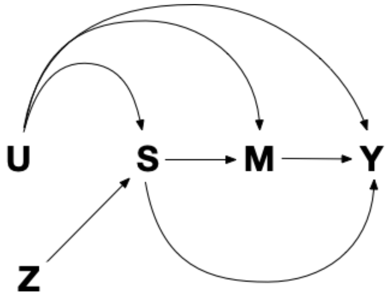


(c) Two confounders with $M$ unconfounded by $U$

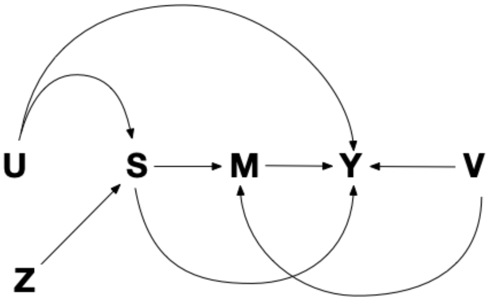


(d) Two confounders with $M$ confounded by $U$

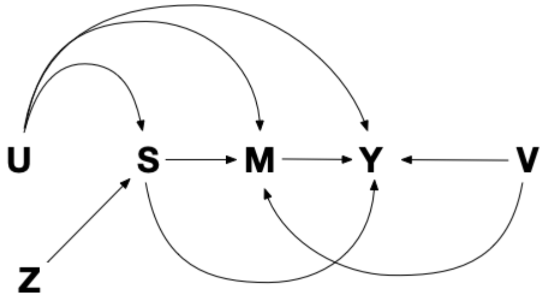

Supplement: Supplementary file 1 — Supporting Information S1 [file HEC-35-742-s001.pdf]
